# Challenges in the Classification of Cardiac Arrhythmias and Ischemia Using End-to-End Deep Learning and the Electrocardiogram: A Systematic Review

**DOI:** 10.3390/diagnostics16010161

**Published:** 2026-01-04

**Authors:** Edgard Oporto, David Mauricio, Nelson Maculan, Giuliana Uribe

**Affiliations:** 1 Facultad de Ingeniería de Sistemas e Informática, Universidad Nacional Mayor de San Marcos, Lima 15081, Peru; dmauricios@unmsm.edu.pe; 2 Ingeniería de Sistemas-Ciencias de la Computación y Matemática Aplicada, Centro de Tecnologia & Centro de Ciências Matemáticas e da Natureza, Ilha do Fundão Campus, Universidad Federal de Río de Janeiro, Río de Janeiro 21941-617, Brazil; maculan@cos.ufrj.br; 3 Instituto Nacional de Salud del Niño, Lima 15083, Peru; guribeg@insn.gob.pe

**Keywords:** end-to-end deep learning, challenges, classification, arrhythmia, ischemia, electrocardiogram

## Abstract

**Background**: Cardiac arrhythmias and ischemia are increasingly problematic worldwide because of their frequency, as well as the economic burden they confer. **Methods**: This research presents a systematic literature review (SLR), based on the PRISMA 2020 statement, that looks into the difficulties in their classification using end-to-end deep learning (DL) techniques and the electrocardiogram (ECG) from 2019 to 2025. A total of 121 relevant studies were identified from Scopus, Web of Science, and IEEE Xplore, and an inventory was created, categorized into six facets that researchers apply in DL studies: preprocessing, DL architectures, databases, evaluation metrics, pathologies, and explainability techniques. **Results**: Fifty-three challenges were reported, divided between end-to-end DL techniques (15), databases (18), pathologies (9), preprocessing (2), explainability (8), and evaluation metrics (1). Some of the complications identified were the complexity of pathological manifestations in the ECG signal, the large number of classes, the use of multiple leads, comorbidity, and the presence of different factors that change the expected patterns. Crucially, this SLR identified 18 new issues: four related to preprocessing, three related to end-to-end DL, one to databases, one to pathologies, four to metrics, and five to explainability. Particularly notable are the limitations of current metrics for assessing explainability and model decision confidence. **Conclusions**: This study clarifies all these limitations and provides a structured inventory and discussion of them, which can be useful to researchers, clinicians, and developers in enhancing existing techniques and designing new ECG-based end-to-end DL strategies, leading to more robust, generalizable, and reliable solutions.

## 1. Introduction

Cardiovascular diseases (CVDs) cause the most deaths and disabilities worldwide [1]; CVD-related fatalities have increased, going up from 12.3 to 19.4 million between 1990 and 2021, and in the U.S., someone dies of CVD every 34 s—a situation that is extremely alarming, totaling about 2580 per day [2]. Thus, they are an overwhelming burden on the health system, with estimates showing that deaths caused by CVDs will reach 20.5 million in 2025 and 35.6 million in 2050 [3]. There are several causes behind this high incidence of the condition, with the most important being hypertension, high low-density lipoprotein cholesterol, and hyperglycemia [4]. Unhealthy diet and smoking as behavioral risk factors, and overweight and obesity, which afflict 59% of adults across the globe, worsen the scenario [5,6]. Air pollution with fine particulate matter is among the environmental risk factors contributing most to the disease burden [7]. In addition, a critical shortage of specialists hampers timely diagnosis and appropriate treatment. Some of the most common diseases linked to CVDs are cardiomyopathies, heart failure, coronary heart disease, and arrhythmias [8]. They are all serious conditions and require accurate diagnosis and effective clinical management [9]. This research is going to study the last two pathologies due to their healthcare relevance and challenge of classification using the DL models.

Cardiac arrhythmias represent disturbances of the regular heart rhythm, and cover an extensive range of pathologies. Atrial fibrillation (AF) is the most predominant arrhythmia among adults, constituting a serious threat that causes significant morbidity and mortality [10], with AF now considered a global epidemic [11]. AF affects approximately one in three to five people after the age of 45, with a lifetime risk from that age onward. The current burden of AF increased from 34 to 59 million cases between 2010 and 2019, with 0.34 million deaths in 2021 [12]. Age is the leading factor for AF, where cases rise significantly after age 65 [11]. Ischemic heart disease, on the other hand, is a group of conditions resulting from partial or complete blockage of coronary blood flow, caused by the accumulation of plaques of fatty materials and cholesterol in the arterial walls. Coronary ischemia continues to be the predominant cause of mortality globally [4], with cases increasing from 5.37 million in 1990 to 8.99 million in 2021; furthermore, current projections suggest it will dominate mortality statistics until 2050 [2,3]. Myocardial damage is irreversible if ischemic heart disease is permitted to develop or continue; thus, effective management of underlying risk factors is essential not only to reduce the incidence of myocardial infarction but also to improve long-term prognosis.

Cardiac arrhythmia and ischemia classification (CAIC) based on analysis of the electrocardiogram (ECG) is not straightforward given the complicated waveform and highly dynamic behavior of the ECG. Classic machine learning techniques require considerable preprocessing and feature engineering by hand. As a result, they are not suitable for scaling, capturing complex patterns, and reproducibility, to name a few. Meanwhile, end-to-end DL methods can allow the raw signal to be directly analyzed, or with minimum preprocessing, and automatically extract features, thereby facilitating automation; in addition, they also aid in handling huge amounts of data. As a result, diagnosis and early detection are improved as well as treatment [13]. Research has revealed the potential of DL models in portable devices and telehealth, helping to improve timely diagnostic access in rural regions and making the spectrum of healthcare more equitable [14,15,16]. Accordingly, end-to-end DL techniques are examined as they can automate the full classification with great potential.

Among the studies on CAIC using ECG and end-to-end DL, ref. [17] employed an end-to-end CNN–Improved Bidirectional LSTM network for arrhythmia classification with the MIT-BIH Arrhythmia Database and the MIT-BIH Atrial Fibrillation Database, achieving 97.85% accuracy and a 97.95% sensitivity. Ref. [18] applied ResNet with an attention mechanism to detect six arrhythmias, reporting an F1-score of 88% and a specificity of 97%. Ref. [19] used a CNN combined with GRU for myocardial infarction detection with the PTB-XL Database, achieving both an accuracy and sensitivity of 99.1%. Ref. [20] utilized CNN, Bi-LSTM, and Bi-GRU in an end-to-end approach to categorize multiple arrhythmias, obtaining an accuracy of 98.55% and a recall of 0.9831. Finally, ref. [21] developed a CNN–Transformer model with dual-view and an external attention mechanism, end-to-end, using the CPSC-2018 database to detect six arrhythmias and ST-segment changes, reaching an 0.85 F1-score and an 0.863 accuracy rate.

CAIC through DL techniques and ECG still faces significant challenges, including low data quality, high variability in ECG signals, lack of model explainability, the contamination of the signals with noise and several artifacts, and the underrepresentation of certain pathologies in training datasets [13]. The widespread use of end-to-end DL architectures in the healthcare domain faces limitations because of the above problems. But this raises an important question. What difficulties does CAIC encounter when using end-to-end DL? In-depth knowledge behind every challenge is key to building novel algorithms or enhancing existing algorithms to improve performance and implementations, which will ultimately help with trust and user uptake [18].

Since 2019, multiple systematic reviews have examined the use of artificial intelligence for cardiac pathology classification with the ECG; however, these reviews typically address a broad spectrum of techniques, including traditional machine learning. To date, few systematic reviews have provided a comprehensive analysis of end-to-end DL pipelines, nor highlighted the specific challenges they face in classifying arrhythmias and ischemia using a rigorous methodology approach.

Because studies on the classification of arrhythmias and ischemia with end-to-end DL pipelines reflect difficulties across various aspects and methodologies, a systematic review becomes necessary to integrate these findings, identify patterns, and assess their impact on the models.

Unlike previous systematic literature reviews, this study focuses only on end-to-end DL architectures and the critical systematization of the difficulties they face in classifying arrhythmias and ischemia. By applying this particular perspective to the literature published between 2019 and 2025, our work provides a complementary and more focused contribution than existing reviews.

The primary goal of this review is to ascertain and scrutinize the techno-methodological barriers to the use of end-to-end DL models with ECG. Accordingly, several articles published from 2019 to 2025 formed the basis of this systematic literature review (SLR). Clinicians, biomedical companies, and researchers can use the findings to refine current algorithms, implement them into clinical practice, and develop more optimized and reliable medical applications. These findings also hint at new avenues of research.

This research aims (a) to provide an overview of heart functioning as well as cardiac arrhythmias and ischemia with respect to their causes, classification, diagnosis, and aspects of study; (b) to present an inventory of CAIC research with end-to-end DL and ECG with respect to their preprocessing approaches, end-to-end DL models, databases, cardiac pathologies, evaluation metrics, and explainability approaches; and (c) to provide an inventory of challenges in CAIC with end-to-end DL and ECG with respect to those already reported in the literature and those not reported yet.

This study is structured into five sections, described below. Section 2 contains a tutorial on cardiac function, as well as characteristics and patterns defining cardiac arrhythmias and ischemia on the ECG waveforms. Section 3, Section 4 and Section 5 contains a systematic review of CAIC using end-to-end DL architectures and the ECG. The challenges of utilizing end-to-end DL models for CAIC are discussed in Section 6. Section 7 interprets the results. Lastly, Section 8 is dedicated to the conclusions.

## 2. Background

### 2.1. Electrical Control of Heart Pumping

The heart sends oxygenated blood to the body and deoxygenated blood to the lungs. In terms of structure, it has four chambers (i.e., two atria and two ventricles), valves, arteries, veins, and myocardium [22]. The heart goes through diastole, the period where the heart muscle relaxes and receives blood, and systole, which is when the heart contracts and pushes blood to the lungs and body. The cardiac conduction system controls these phases. The electrical impulses start in the sinoatrial node, travel through the atria where the electrical activity is delayed for a short time at the atrioventricular node, and then are transmitted through the bundle of His (AV bundle) and Purkinje fibers, causing the contraction of the ventricles [23].

### 2.2. The ECG and Its Leads

**Figure 1:**
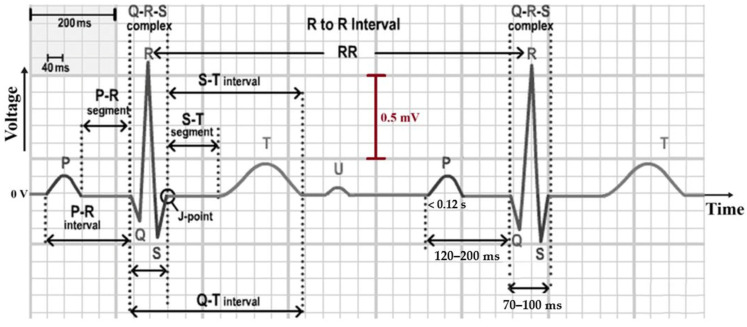
Typical ECG signal of a healthy individual. Adapted from [27].

ECG signals are recorded using cutaneous electrodes and include waves, segments, and intervals [24]. The P wave refers to atrial depolarization, the QRS complex is for ventricular activation, while the T wave is for ventricular repolarization. The typical value of signal span is 2 mV, and the duration of a cardiac cycle is about 1 s. This varies in different individuals and conditions [22,25]. A typical ECG waveform is presented in Figure 1. Moreover, the standard 12-lead ECG records the heart’s bioelectrical activity through bipolar limb leads (I–III), precordial leads (V1–V6), and augmented unipolar limb leads (aVR, aVL, and aVF). Each lead provides a distinct view on cardiac regions, and signals from aVR, aVL, and aVF are derived algebraically from leads I, II, and III [26].

### 2.3. Arrhythmias: Causes and Classification

Arrhythmias are disturbances in the heart’s electrical activity that manifest as irregularities in rhythm, rate, or waveform; their causes range from underlying cardiac disease to stress, drug exposure, or genetic predisposition [28]. They are commonly classified by the site of origin—ventricular, supraventricular, atrioventricular junction, or sinoatrial node—and by the mechanism, which involves either abnormal impulse formation or impaired conduction. Disorders of impulse formation may result from triggered activity or irregular automaticity, producing conditions such as sinus tachycardia, bradycardia, ectopic rhythms, pauses, torsades de pointes, or digitalis-induced arrhythmias [24]. Conduction abnormalities, in turn, involve blocks or delays in propagation, and are divided into non-reentrant conduction blocks—including sinoatrial, atrioventricular, and bundle branch blocks—as well as aberrant supraventricular complexes and reentrant mechanisms, which underlie sinus reentrant tachycardia, atrial and nodal reentrant tachycardias, atrioventricular reentrant tachycardias with accessory pathways, atrial flutter, atrial fibrillation, and ventricular tachycardia or fibrillation [26].

### 2.4. Ischemia: Causes and Consequences

Cardiac ischemia arises from reduced myocardial perfusion caused by partial or complete obstruction of the coronary arteries. Prolonged ischemia causes tissue necrosis, whereas transient episodes can produce reversible lesions with variable outcomes [29]. The characteristics of myocardial injury depend on the affected artery, and ECG leads provide spatial information to localize the compromised region [23].

### 2.5. Related Research

Several systematic reviews have addressed denoising techniques for cardiovascular signal analysis. Ref. [30] examined 198 studies published between 2017 and 2023, emphasizing database availability and the classification of eight cardiovascular disease types. Ref. [31] focused on 112 studies from 2020 to 2024, highlighting advanced denoising methods for personalized ECG diagnosis and the challenges of inter-patient variability. Ref. [32], in a review of 368 articles up to 2022, identified major trends and research opportunities in arrhythmia classification, with particular attention to databases and commonly used denoising models. Ref. [13] provided an overview of 78 studies from 2017 to 2023, categorizing deep learning architectures that achieved over 96% accuracy in arrhythmia detection, while also offering medical background and methodological guidance. The systematic reviews together represent the course of evolution of denoising techniques, the role of databases, and the diversity of methodologies in the classification of arrhythmias.

Beyond those systematic reviews, several more general studies have looked at deep learning and artificial intelligence in cardiology. According to [33], randomized controlled trials were reviewed to evaluate clinical effectiveness and the applications across arrhythmias, and ischemia and structural heart disease were examined; the authors argued that trained DL strategies show promise in controlled settings, yet noted that real-world implementation will face challenges owing to commonly seen variations in datasets, a lack of standardization, and the need for multicenter validation. In a comprehensive overview of the literature, that is the study [34] which analyzed 200 studies published from 2020 to 2024, we find the use of AI in cardiology. This covers general clinical practice, including the prevention and intervention for arrhythmias, ischemia, and valvular disease. Ref. [35] concentrated on ECG analysis with AI, especially deep learning applied to arrhythmia detection and prediction, myocardial infarction, and other cardiac conditions; the research also raised ethical issues and problems around lack of interpretability. These reviews highlight the clinical potential, as well as the methodological and ethical challenges, of the use of AI in cardiology.

Extending this perspective, ref. [36] surveyed journal and conference articles published between 2019 and 2024, focusing on transformer-based and large language model methodologies for ECG diagnosis. The study provides a hierarchical classification of the reviewed methods, compares categories of approaches, and highlights research gaps along with future directions.

### 2.6. Aspects of Study

**Figure 2:**
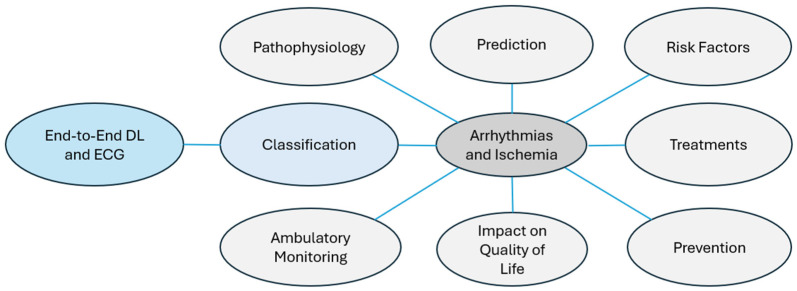
Changes in the ECG signal associated with each type of ischemia. Adapted from [22].

This study focuses on end-to-end DL techniques for automated CAIC using the ECG; however, various other aspects of cardiac arrhythmias and ischemia exist, which are shown in Figure 2 and described below.

- Pathophysiology: The study of biological processes that alter heart rhythm or blood flow. For example, an imbalance between the sympathetic and parasympathetic systems can lead to arrhythmias [37].
- Classification: Techniques for identifying cardiac arrhythmias and ischemia, may or may not be ECG-based. Visual inspection [29], echocardiography [38], end-to-end DL [17], and conventional machine learning [39] are some examples.
- Ambulatory Monitoring: Continuous tracking with portable devices to detect cardiac events in real time, such as Holter monitors integrated with IoT technology [40].
- Risk Factors: Identification of conditions that predispose individuals to cardiac diseases. For instance, obesity increases the risk of AF [41].
- Prevention: Measures aimed at minimizing cardiac arrhythmias or ischemia through modification of lifestyle or early intervention. For example, regular physical activity minimizes the risk of cardiac infarction [42].
- Treatments: Therapies designed to avoid or control arrhythmias and ischemia; for example, catheter ablation, which eliminates tachycardia [43].
- Impact on Quality of Life: Assessment of how heart disease affects emotional, physical, and social well-being. For example, patients recently diagnosed with ischemia may suffer from chronic anxiety [44].
- Prediction: Use of sophisticated algorithms to anticipate the occurrence of critical conditions. For example, ref. [45] proposed a fuzzy DL model to predict cardiac arrhythmias at their outset.

## 3. Materials and Methods

This section presents an SLR on CAIC through end-to-end DL as per the methodology and for planning, execution, results, and analysis. This study uses stringent inclusion criteria to focus exclusively on end-to-end deep learning architectures, in contrast to previous reviews. Furthermore, we conduct a systematic analysis of the methodological challenges in 6 essential components, namely, preprocessing, databases, pathologies, end-to-end DL models, evaluation metrics, and explainability. This methodological perspective is applied to the literature published in the period from 2019 to 2025. It highlights the scope of our SLR. Furthermore, it differentiates our SLR from existing SLRs.

### 3.1. Methodology

The 2020 PRISMA Statement [46] defines the article selection procedure for this SLR to ensure transparency and rigor (see Supplementary Materials, Tables S10 and S11). This strategy is consistent with recent systematic reviews; for instance, refs. [47,48] supply detailed surveys of the uses of artificial intelligence in cardiovascular disease diagnosis using the ECG. The specifications proposed by [49] for software engineering studies have been adopted as well. According to guidelines, the four phases used for SLRs on DL for CAICs, like [32,50,51], are explained below.

- Planning: At this stage of the research protocol, investigators write draft a research protocol that contains the research questions and article search and selection procedure. This includes journal source selection, date selection, search strings, and inclusion and exclusion criteria.
- Execution: The protocol is utilized to select relevant articles addressing the formulated research questions and answering them.
- Results: Determination and presentation of statistics on the selected articles, including trends, quality, and distribution.
- Analysis: The researchers will be required to analyze the research questions formulated at the planning stage.

### 3.2. Planning

To answer the research question on the difficulties of CAIC with end-to-end DL and ECG, the guiding question was as follows: how is CAIC performed with end-to-end DL and ECG? To answer this, a search for journal publications was conducted in the Scopus, Web of Science, IEEE Xplore, and PubMed databases, covering the period from 2019 to 2025. The search string used was as follows: [(diagnosis OR algorithm OR detection OR classification) AND (“cardiovascular diseases” OR “coronary events” OR arrhythmia OR cardiac OR “heart attack” OR “myocardial infarction” OR ischemia OR “atrial fibrillation”) AND (ecg OR electrocardiogram) AND (“deep learning” OR cnn OR rnn OR lstm OR gru OR transformer OR autoencoder)]. This string was applied to “Title–Abs–Key” in Scopus, “Topic” in Web of Science, and “Title–Abstract” in PubMed. After identifying the scientific articles, the selection criteria summarized in Table 1 were applied to determine the eligible studies.

### 3.3. Execution

**Figure 3:**
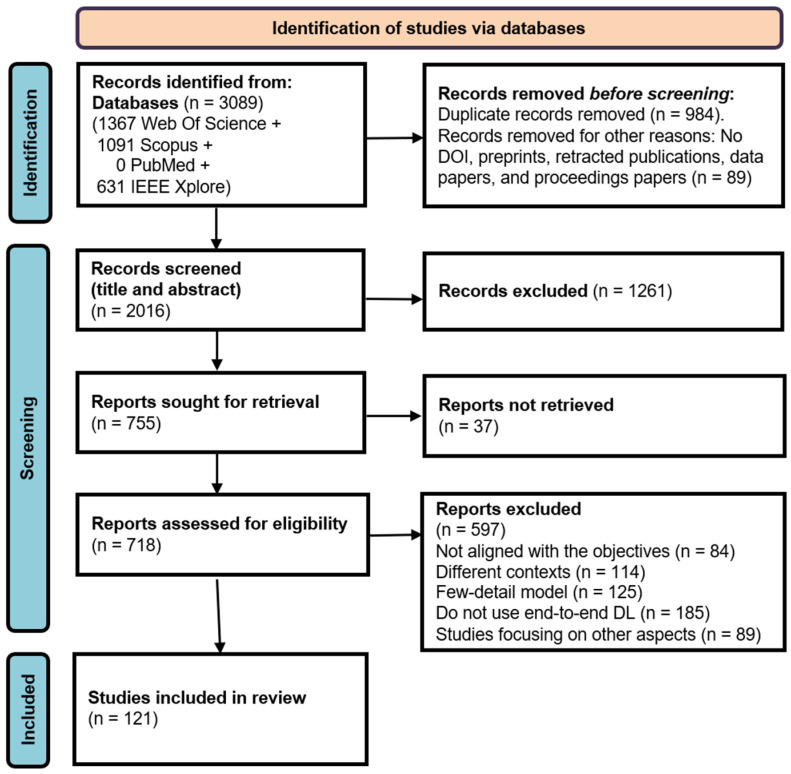
PRISMA selection process used for the literature review [52].

After applying the search strategies, 3089 studies were selected. Next, we systematically reviewed these studies; the screening and selection process is summarized in Figure 3 and was conducted using the inclusion–exclusion criteria shown in Table 1. An Excel file was employed for the exercise to record the selected studies and capture important data like title, author, journal, DOI, and so on.

Initially, a total of 1073 studies were eliminated, including duplicates and other removals, resulting in 2016 articles. During stage two, titles and abstracts were screened, and 1261 studies that failed to satisfy the eligibility criteria were discarded, leaving 755 studies. In stage three, 718 full-text articles were retrieved. In the final stage, a full-text reading of articles was performed to find out those whose contribution is relevant to this review, resulting in 121 articles. Finally, these studies were rigorously analyzed, avoiding subjective interpretations, to find answers to the research question.

## 4. Results

### 4.1. Potential Articles

In total, 2016 potential articles were identified, and 121 were ultimately selected, accounting for 6% of the total (see Table 2). Although no articles were retrieved, PubMed was included in the search strategy given its relevance as a leading clinical database. Additionally, its inclusion ensured comprehensiveness and avoided potential bias due to a limited selection of sources.

### 4.2. Publication Trends

**Figure 4:**
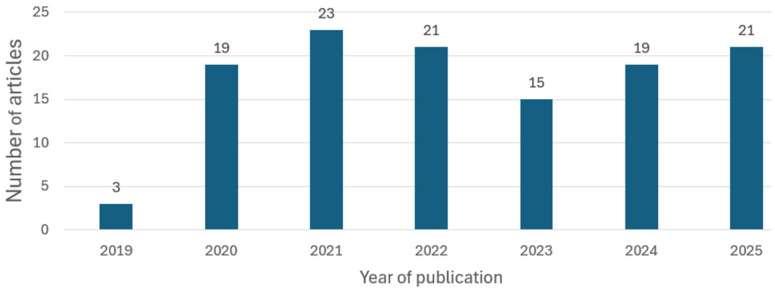
Distribution of articles by publication year.

Figure 4 presents the distribution of the selected articles for the period 2019–2025. A significant increase in research output is observed starting in 2020, which corresponds to the emergence of the first relevant end-to-end deep learning (DL) studies in ECG around 2018, with pioneering contributions of [155]. The volume of publications remained relatively consistent through 2025. This trend reflects sustained activity in this field.

### 4.3. Selected Articles by Journal Quality Factor

**Figure 5:**
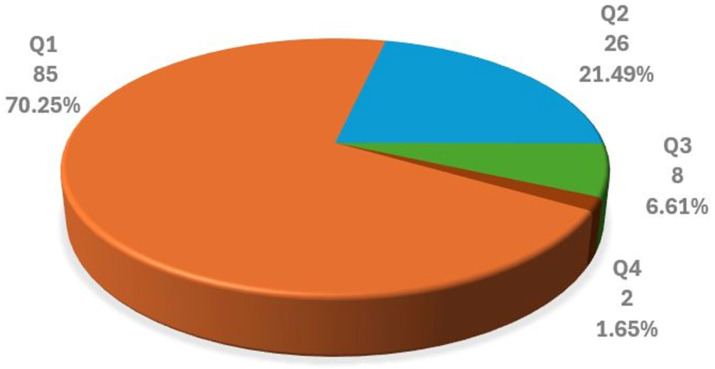
Distribution of articles by quartiles.

Regarding journal quality, 70.25% (*n* = 85) of the selected articles were published in Q1 journals and 21.49% (*n* = 26) in Q2 journals. In total, 91.74% (*n* = 111) of the 121 selected articles appeared in the top two quartiles, underscoring their quality (see Figure 5).

### 4.4. Selected Articles by Journal

**Figure 6:**
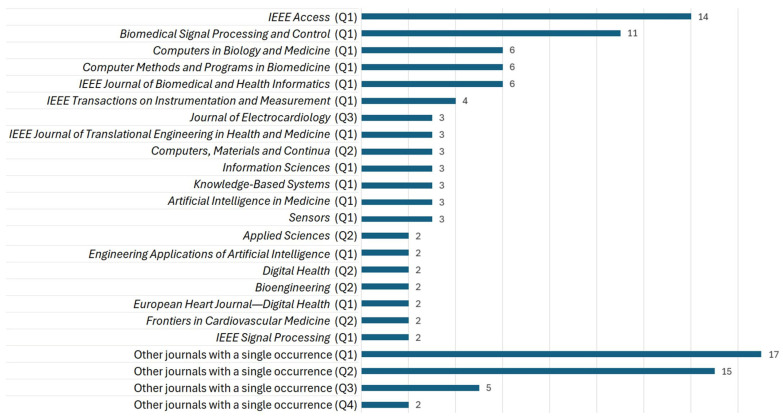
Counts of selected articles by journal.

Figure 6 presents the distribution of the chosen articles by journal; those with only one article are grouped under “Other journals with a single occurrence”.

## 5. Analysis

This section responds to the research question outlined in Section 3.2 through the following sub-questions:

- RQ1: What preprocessing techniques are applied to ECG signals?
- RQ2: What end-to-end DL techniques are employed for feature extraction and CAIC from ECG?
- RQ3: Which databases are used to train and validate end-to-end DL algorithms?
- RQ4: What types of cardiac arrhythmias and ischemia are classified by the algorithms?
- RQ5: What metrics are used to evaluate the effectiveness of end-to-end DL algorithms?
- RQ6: Which techniques are used to explain the results of ECG-based CAIC using end-to-end DL?

### 5.1. RQ1

**Figure 7:**
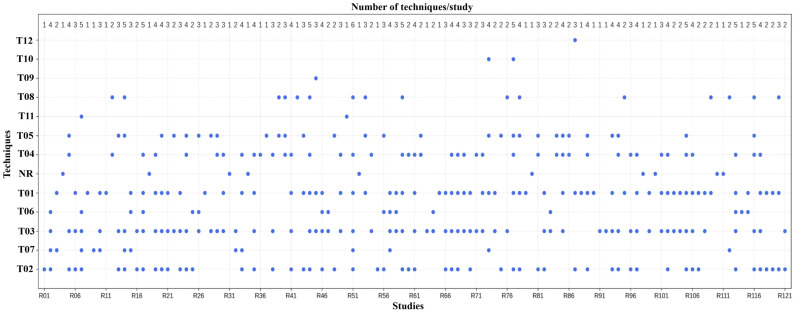
Preprocessing techniques used by each author (NR: not reported).

Twelve types of techniques were identified for preprocessing ECG signals prior to their use in end-to-end DL models (see Table 3). Among these, the most recurrent during the training, validation, and inference phases were segmentation, amplitude normalization, and noise and artifact removal, owing to their direct impact on data quality and model stability. By contrast, techniques such as resampling, structural data adjustment, class balancing, and advanced cleaning were less frequently employed (see Figure 7). These were primarily applied during the model construction phase to obtain suitable data because the final model generally operated on signals that already conformed to the required input format and did not require further modification or class-distribution adjustment.

Figure 7 illustrates the distribution of preprocessing techniques across the studies. The left axis shows the techniques’ identifiers, while the top axis shows how many techniques are employed in each study.

Having outlined the overall distribution and relevance of preprocessing techniques (Table 3, Figure 7), we now describe each category and specific techniques in detail (see Table 4). Additional information can be found in Supplementary Tables S7–S9.

- Techniques T01: Noise and Artifact Removal
  The methods used in this category are used for preprocessing the ECG signals in order to improve their quality. Wavelet-based methods rely on multi-resolution decomposition to separate waves and suppress noise components. Digital filters (Butterworth, band-pass, and notch) are used to suppress other frequencies, such as baseline wander and power-line noise. LOESS, moving average, and Non-Local Means (NLM) smoothing are statistical methods that use local signal similarity to suppress noise. To minimize amplitude changes, normalization methods are applied (sliding window). Furthermore, to discard residual noise, a thresholding strategy is employed (such as a hard threshold or wavelet threshold), discarding coefficients that went below a defined level. The purposes of artifact removal, baseline wander correction, high-frequency noise suppression, and residual noise removal represent complementary approaches to the common objective of enhancing ECG signal quality. In their studies, authors have labeled the techniques differently, but they are all aimed at solving the same noise and distortion problems in ECG preprocessing. In our corpus, 42 studies used some processing for noise or artifact removal.
- Techniques T02–05
  To ensure uniformity of the ECG signal amplitude, segmentation of the temporal structure on the recordings, and the harmonizing of sampling rates of the various datasets, preprocessing techniques T02–T04 were implemented. Normalization methods (T02) include Z-score scaling, Min–Max scaling, and unit variance adjustment to avoid varied amplitude ranges in the model. Windowing approaches (T03) segment signals into fixed-length segments of size 1.5–60 s using either a single window or multiple windows, with or without overlap, for local analysis and feature extraction. Resampling techniques (T04) modify the temporal resolution of a signal through downsampling or upsampling, aiming to create uniform sampling frequency data aligned in time and to process heterogeneous sources. These techniques enhance signal comparability and model compatibility, and were reported across a wide range of studies.
  The techniques under T05 deal with forcing identical signal duration and identical structure prior to the model input. The techniques used include zero-padding, cropping, trimming, replication, segmentation, and resampling. These methods were applied to obtain fixed-length signals of length 2.5 s to 2 min and sample length 4096 and 9000, respectively. Short recordings are padded or duplicated, while long recordings are cropped or split into overlapping recordings. These adaptations ensure that model architectures can leverage batch processing, allowing consistent feature extraction from various datasets. While the techniques vary across studies, they all attempt to bring the length and format of definitions to a more acceptable level to facilitate feature extraction and model training. According to Table S8, these approaches were analyzed in 25 papers.
- Techniques T06–T12
  In total, 41 techniques were identified in categories T06–T12, reported across 34 studies: 14 techniques in T06, 11 in T07, 11 in T08, 2 in T12, and 1 each in T09–T11.
  Techniques to balance classes (T06) are shown in Table 4; oversampling methods such as SMOTE and GAN, as well as downsampling and replication, are countermeasures to improve class balance. Techniques of data cleaning (T07) are used to remove redundant and missing values, noise, indistinct segments, duplicate values, and anomalous signal parts to add accuracy to the input. The techniques of augmentation (T08) apply operations such as cropping, jittering, warping, and noise insertion to diversify data and limit overfitting. Several less-often-reported categories serve specialized preprocessing roles. Overall, the objective of these techniques is to improve data quality, balance classes, and increase variability.

### 5.2. RQ2

The classification of 121 DL techniques into seven families is shown in Table 5. The seven families put forward complementary techniques that should achieve optimal results on ECG data. Also, we can see the trend in use of each technique family. CNN models prevail in the literature, owing to their ability to extract morphological features from complex ECG signals across one or more leads. RNN-based modeling may be rare when compared to the above-mentioned models, but have their uses too. They can model the rhythm of a sequential dependence quite well. Hybrid CNN-RNN frameworks combine CNNs and RNNs that enable the use of spatial and temporal representations. Despite being less so, the transformer-based model introduces scalability and parallelization indicating a promising way forward in long-range dependencies. Increasingly adopted models with enhanced attentional abilities emphasize the importance of interpretability and the dynamic weighting of features in clinical applications. Generative and contrastive methods are useful for representation learning and improving data use efficiency, especially when labels are scarce. Last but not least, the custom/ensemble/NAS models show the architectural optimization and deployment efficiency pursuit.

As a whole, these families exemplify methodological diversification: CNNs remain the backbone, but attention mechanisms, generative paradigms, and automated design search are becoming increasingly important. This comparative lens not only highlights contributions of different families but also their interplay to shape end-to-end ECG analysis. Supplementary Table S1 includes a full list of all 121 techniques plus references for complete transparency and traceability.

### 5.3. RQ3

To assess the 52 databases referenced in the studies reviewed, we must first assign standard abbreviations to the cardiac conditions that cause arrhythmic and ischemic effects since each database states which condition it will cover. Supplementary Table S2 contains the full list of abbreviations. The databases serve multiple functions in model development and evaluation, such as training, validation, testing, and inference. Most of the reviewed studies relied on more than one data source because a single database rarely provides sufficient diversity in terms of pathological classes, patient age ranges, recording devices, or annotation quality. The 17 databases most frequently used across studies—together with the cardiac conditions they cover and the studies in which they were applied—are detailed in Supplementary Table S3, while the 35 databases used in a single study are listed in Supplementary Table S4.

**Figure 8:**
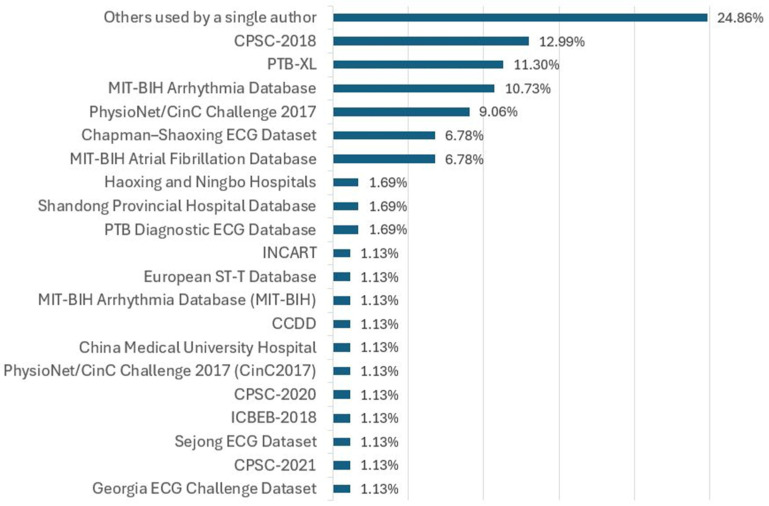
Percentage of database usage.

Figure 8 presents the statistics on the use of the 52 databases across the selected studies. In total, these databases were used 163 times. The six most-often-employed databases—CPSC-2018, MIT-BIH, PTB-XL, CinC2017, AFDB, and Chapman–Shaoxing ECG Dataset—account for 57.64% of all instances of use. By contrast, the 35 databases used only once represent 21.47% of the total usage.

Table 6 complements the information in Supplementary Tables S3 and S4 by detailing the key characteristics of the 52 identified databases. Supplementary Table S5 provides download links for the 28 public databases.

### 5.4. RQ4

**Figure 9:**
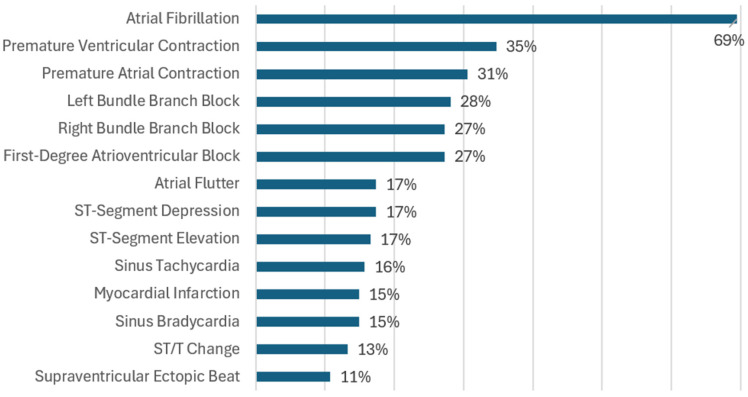
Percentage of studies per pathology (only the 14 most frequent are shown).

In total, 163 types of cardiac arrhythmias and ischemia were identified; these are listed and abbreviated in Table 6. Supplementary Tables S3 and S4 indicate the databases and studies in which they appear. Figure 9 illustrates the percentage of studies addressing the 14 most-often-investigated cardiac pathologies out of the total selected studies. Among them, AF was the most studied, appearing in 84 of the 121 studies (69%). It should be noted that some studies included more than one pathology.

The articles corresponding to the pathologies shown in Figure 9 were identified from Tables S3 and S4 and are summarized in Supplementary Table S6 according to usage count and references.

### 5.5. RQ5

Table 7 presents the 11 metrics employed in the studies analyzed in this review. Each metric is accompanied by a precise definition and the recommended scenario for its application.

**Figure 10:**
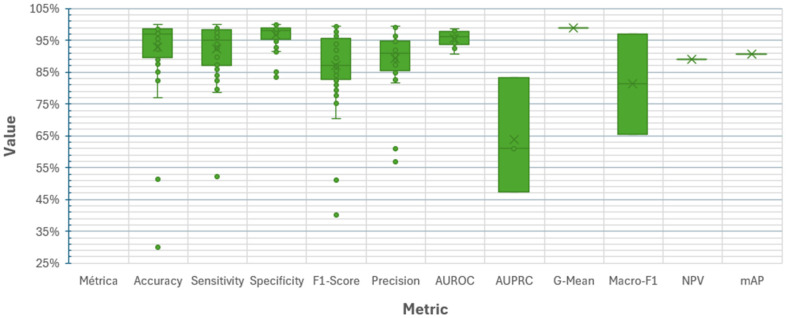
Distribution of performance metric results in end-to-end DL models for CAIC with ECG.

Figure 10 presents the distribution of results by performance metric for the 121 end-to-end DL models for CAIC analyzed in this review. These results of each metric are not necessarily comparable because the studies relied on different databases that vary in the number of classes, the degree of class imbalance, and the allocation of data for training, validation, or testing. The F1-score, precision, accuracy, and sensitivity metrics show high values (above 95%) but with dispersion. AUROC and specificity also achieve high values, though with low dispersion. By contrast, AUPRC and Macro-F1 show more scattered values, generally below 90%. Finally, G-Mean, NPV, and mAP were each reported in only one study, with values around 95%.

### 5.6. RQ6

Interpretability is the inherent ability of a model to be understood, both in terms of its internal logic and the way it generates results. This is characteristic of so-called white-box models, like Support Vector Machines or Linear Regression, whose structure and operation are transparent. Unlike ML models, DL models can be seen as black boxes. This is due to their complex architectures that have thousands or millions of trainable parameters between any two layers. As such, it is difficult to ascertain the logic behind the inference made by DL models. Incorporating explainability mechanisms helps uncover or clarify the decision-making of the models. Explainability can be applied post hoc, that is, externally after training, using techniques such as weighted activation maps (Grad-CAM). Alternatively, it can be embedded directly into the model’s design, as in architectures based on attention mechanisms. In either case, the explanations do not fully eliminate the opacity of DL models but instead provide a partial—yet valuable—approximation of the reasoning behind their outputs.

Table 8 presents the 23 explainability techniques identified across 43 selected studies, along with brief descriptions and their type of explainability.

## 6. Challenges of CAIC End-to-End DL and the ECG

The challenges discussed in this section refer to the barriers and limitations that hinder the development of CAIC through end-to-end DL techniques and the use of ECG signals, as well as their integration into hospital systems. These challenges were identified using the method described in Section 6.1, with its execution detailed in Section 6.2, and the results—which outline the specific challenges—presented in Section 6.3.

### 6.1. Method

The method used to identify challenges in CAIC through end-to-end DL and ECG comprised five phases:

- Phase 1. Study Inventory: Relevant information on CAIC using end-to-end DL and ECG was collected from the specialized literature.
- Phase 2. Determination of the Purpose of each Analysis Aspect: The purpose of each analysis aspect was derived from its definition.
- Phase 3. Inventory of Challenges in the Analysis Aspects: A comprehensive review of the challenges reported in the collected studies was conducted for each analysis aspect.
- Phase 4. Identification of Unaddressed Challenges: Gaps not addressed in the literature were determined by comparing the inventory of challenges with the stated purposes of the analysis aspects.
- Phase 5. Discussion of Findings: The challenges identified in the previous phases were discussed, highlighting their implications for future research and the development of CAIC solutions. This phase is presented in Section 5.

### 6.2. Development

In Phase 1, described in Section 3, 121 relevant studies on CAIC using end-to-end DL and ECG were identified. These studies formed the basis for compiling inventories across the following aspects: preprocessing techniques, end-to-end DL methods, databases used, cardiac pathologies studied, evaluation metrics, and explainability approaches. These aspects constitute the analytical dimensions of this review. Because challenges in these areas directly affect the development and implementation of end-to-end DL models for CAIC with ECG, Phase 2 established the purposes of each aspect, which are presented in Table 9.

In Phase 3, fifteen difficulties were identified for end-to-end DL techniques, as reported in 53 of the selected studies (Table 10). Additionally, Table 11, Table 12, Table 13, Table 14 and Table 15 detail the difficulties associated with each of the remaining five analysis aspects. 

Eighteen database-related challenges were identified, explicitly reported in 72 of the selected studies (Table 11). The effects of these challenges on DL model performance are also detailed.

**Table 11:** Challenges related to databases.

ID	Difficulty	Effects	References
D16	Lack of large, well-annotated databases for portable devices	Limits generalization of models trained on standard clinical ECGs. Makes it difficult to capture artifacts specific to ambulatory use.	[128]
D17	Imbalance between positive classes or between positive and negative classes	Biases the model toward the majority class and reduces performance for clinically important conditions.	[17,18,20,54,55,56,57,58,59,62,64,67,68,69,71,73,74,75,76,80,81,82,83,85,86,88,89,91,92,93,95,98,101,102,107,113,116,118,119,120,121,123,125,126,129,130,131,132,133,136]
D18	Scarcity of sufficiently large, diverse, and annotated databases	Weakens robustness and generalization to new clinical contexts. Leads to overfitting and hinders training of large or complex models.	[17,21,40,53,58,59,62,66,79,80,88,90,93,96,99,102,107,114,121,122,128]
D19	Lack of data standardization or quality	Requires more diverse and labor-intensive preprocessing due to incompatibilities. Complicates cross-validation and benchmarking.	[18,21,57,58,78,79,83,91,126]
D20	Underrepresentation of diverse populations	Introduces bias and limits applicability to generalized clinical use.	[17,18,57,72,92,96,116,126]
D21	Restricted access and privacy issues	Complicates data collection, sharing, and use. Prevents external validation and reproducibility.	[18,20,53,58,59,61,74,78,97,98,100,101,104,107,134]
D22	Different sampling rates across databases	Causes loss of information or signal distortion from resampling.	[57,114,116]
D23	Data from a single source or device	Produces bias toward the source device, excessive dependence on calibration, and poor generalization to other datasets. Overestimates model capability and reduces external validity.	[21,57,91,104,106,119,126,127,129,130,131]
D24	Variability among acquisition devices	Creates dependence on specific recording systems, degrades multicenter performance, and hinders cross-validation and benchmarking.	[72,75,78,98]
D25	Limited metadata: age, sex, weight, ethnic origin and population diversity, comorbidities, etc.	Compromises interpretability, fairness, and adaptability of the model to subgroups or vulnerable populations.	[58,59,73,79,80,103]
D26	Limited availability of databases with concurrent pathologies	Prevents training of robust multi-label models and restricts the design of clinically useful models.	[126]
D27	Inconsistent or automated labeling	Leads the model to learn incorrect associations and reduces performance.	[18,55,57,84,89,98,101,102,103,135]
D28	Absence of standardized protocols for acquisition, annotation, and structuring of records in ECG databases	Reduces interoperability between datasets and limits model generalization, transferability, and comparability.	[55,78]
D29	Variability in the number of ECG leads	Reduces model comparability, introduces differences in spatial information, and prevents transfer to devices using different leads.	[70,126]
D30	Dataset coverage restricted to a single pathology	Limits clinical evaluation and prevents training or testing of multi-class and multi-label models.	[110,113]
D31	Inter-database variability in ECG recording duration and quality	Complicates model architecture and joint training, leading to uneven or biased learning.	[92,93]
D32	Fine-tuning	Requires large, high-quality clinical datasets.	[110]
D33	Different recording durations across databases	Increases computational complexity and training difficulty. Performs poorly on long signals where rare or transient events may occur.	[75,80,85,91,95,116,117,127,129]

Table 12 presents the 10 difficulties related to pathologies identified across 48 selected studies, together with their effects on model performance.

**Table 12:** Difficulties in cardiac pathologies.

ID	Difficulty	Effects	References
D34	Pathology similarity	Makes it difficult to extract discriminative features, reducing accuracy in multi-class classification and increasing diagnostic errors. Requires clinically diverse data, precise labeling, and greater model capacity.	[54,58,62,63,71,82,83,88,93,95,97,106,108,113,117,118,123,134]
D35	Comorbidities or multiple concurrent cardiac pathologies	Introduce diagnostic difficulty because one pathology may mask or distort another. Requires well-annotated multi-level databases and more sophisticated architectures capable of learning multiple patterns.	[40,67,82,108,113]
D36	Intra-patient and inter-patient variability	Reduces generalization by blurring physiological and pathological variability. Lowers performance in external cross-validation and limits transferability to new patients.	[18,19,21,53,67,75,78,83,90,93,108,114,120,125,133,136]
D37	Ambiguity in the patterns of certain pathologies	Reduces diagnostic specificity due to inter-class overlap.	[121]
D38	Pathologies with episodic or paroxysmal occurrence	Require long recordings or sequential models; sensitivity is reduced when using short windows.	[17,53,64,68,75,83,87,88,92,93,122,129,133,134]
D39	Subtypes of pathologies	Demand specialized models and finer expert-labeled annotations, increasing complexity and the risk of diagnostic errors.	[72,73,86]
D40	Complex patterns	Require more sophisticated models and larger volumes of annotated data.	[20,21,73,82,83,85,87,92,101,123,128]
D41	Subtle morphological changes in various pathologies	Make detection difficult and require complex models with high resolution or higher sampling rates.	[21,93]
D42	Redundancy of information in the 12-lead ECG	Limits usefulness in deep models, where combinations can be learned automatically, and reduces suitability for portable devices.	[72,131]

Two difficulties were identified in preprocessing techniques (D43 and D44), reported in 33 of the selected studies, and one difficulty (D45) in the metrics used. Table 13 presents these difficulties along with their effects on model performance evaluation.

**Table 13:** Difficulties in preprocessing and metrics.

ID	Difficulty	Effects	References
D43	Presence of excessive or unaccounted noise and artifacts	Increases the risk of losing critical information and reduces model performance in real-world settings.	[17,18,20,58,72,74,75,76,78,79,80,82,85,86,87,90,91,92,93,95,99,114,118,119,120,121,122,125,127,128,129,134,136]
D44	Unrealistic generation of synthetic data	May cause the model to capture non-real features, leading to poor generalization and reduced explainability.	[128]
D45	Absence of standardized metrics for evaluation	Hinders comparison across models; the use of inadequate metrics may obscure poor performance in critical classes.	[18]

Finally, Table 14 presents the eight difficulties related to explainability techniques, identified in 16 of the selected studies. Each difficulty is associated with a specific explainability method.

**Table 14:** Difficulties in the explainability techniques employed.

ID	Technique	Difficulty	Effects	References
D46	T02	Regions highlighted by attention maps do not always match clinically relevant or expected features.	The use of clinical tests has limited acceptance in medical circles as they are neither very useful nor unambiguous.	[59,101]
D47	T03	Does not allow complete reconstruction of the decision-making process; limited in scenarios with high signal variability.	Restricts transparency; the lack of full traceability of the model’s reasoning hinders acceptance and validation in clinical settings.	[129,131,134]
D48	T07	Significant overlap of feature maps; generated maps may not display clinically understandable, relevant, or complete patterns	A reduction in visual clarity and difficulty in identifying the ECG areas influencing the results can lead to ambiguity and low clinical trustworthiness.	[78,84,102]
D49	T09	Explanations can show which areas are important to the model but do not always show areas that the clinician would find important for diagnosis.	Creates misalignment between model logic and clinical reasoning; hinders expert validation and reduces trust in automated decisions.	[60]
D50	T11	Incorrect assignment of relevance to noisy regions.	Produces false conclusions about ECG regions driving predictions; omits significant features, which may mislead analysts and reduce model reliability.	[78]
D51	T13	It is not possible to trace the complete reasoning of the model using these means.	Prevents full causal understanding of decisions; reduces transparency and limits reliability in clinical validation.	[97]
D52	T17	Highlights important regions for the decision without explaining why those regions are relevant.	Obscures the decision-making mechanism, reducing usefulness for clinical analysis or expert validation.	[97,98]
D53	T18	Identifies important ECG regions without establishing correlation with clinical criteria or validating medical relevance.	Limits interpretability; highlighted regions may be technically relevant but not clinically meaningful, reducing their reliability for practitioners.	[61]

### 6.3. Unaddressed Difficulties

In Phase 4, the difficulties reported in the selected studies (Table 10, Table 11, Table 12, Table 13 and Table 14) were cross-referenced with the objectives of the analysis aspects defined in Table 15. This process allowed the identification of 17 difficulties not yet addressed in the literature, which are presented in Table 15.

**Table 15:** Difficulties not yet addressed in studies on CAIC.

ID	Aspect	Unaddressed Difficulties	Justification of the Affected Activity or Feature
D54	Preprocessing	Lack of dynamic normalization adapted to changing clinical contexts	Limits real-time processing of signals that vary due to physiological, technical, clinical, or temporal factors.
D55	Preprocessing	Absence of standards for preprocessing multichannel signals from different devices	Creates compatibility and robustness issues due to technical differences between sources.
D56	Preprocessing	Absence of automatic quality control of signals in real-world environments	Models trained on diagnostic-quality signals fail to generalize to uncontrolled environments.
D57	Preprocessing	Fixed windows misaligned with clinical events	Windows that do not follow physiological or diagnostic boundaries lead to missed detection of brief events.
D58	DL end-to-end techniques	Lack of automatic hyperparameter tuning mechanisms for deep architectures	Reduces efficiency and slows model experimentation and optimization.
D59	DL end-to-end techniques	Integration of self-supervised techniques to pretrain models with limited data	Self-supervised pretraining reduces dependence on large annotated databases.
D60	DL end-to-end techniques	Lack of real-time adaptation to patient changes during prolonged monitoring	Prevents models from adjusting parameters to individual physiological changes, reducing performance.
D61	Database	Creation of synthetic databases to balance minority classes without compromising quality	Rare patterns should be included without degrading model performance.
D62	Cardiac pathologies	Limited consideration of dynamic changes in pathologies	Hampers classification when pathologies evolve dynamically during prolonged monitoring.
D63	Metrics	Limitations of metrics for evaluating explainability and confidence in model decisions	Undermines adoption in medical contexts where explainability is critical.
D64	Metrics	Lack of correlation between computational metrics and clinical outcomes	Disconnect between metrics and clinical decision-making fails to account for clinical risk, diagnostic urgency, or therapeutic utility, hindering objective comparisons.
D65	Metrics	Metrics with limitations for evaluating temporal sequences and real-time performance	Fail to capture event timing or latency, persistence, or continuity. Short events go undetected, and real-time inference cannot be evaluated.
D66	Metrics	Metrics for multi-class classification	Conceal poor performance in minority classes and fail to reflect differences in clinical risk between classes.
D67	Explainability techniques	Lack of visual tools to interpret decisions on long signals (e.g., Holter recordings)	Prevents reliable interpretation of extended ECG records.
D68	Explainability techniques	Lack of explainability adapted to each pathological class	Current techniques do not distinguish between classes with different clinical criteria; an explanation valid for one class may be inadequate for another.
D69	Explainability techniques	Limitations of explanations in multi-label and multi-lead contexts	Visual techniques merge explanatory information, preventing separation of influences by class or ECG lead.
D70	Explainability techniques	Lack of standardized evaluations to assess agreement with expected clinical findings	Reduces the reliability of techniques and prevents comparability across studies.
D71	Explainability techniques	Misalignment between the explanation’s scale and the clinical event’s scale	Explanations highlight very small regions without clinical correlation in duration.

## 7. Discussion

### 7.1. About Preprocessing

Though end-to-end DL models seek to minimize human intervention when conducting ECG analysis, evidence from the 121 reviewed studies shows that preprocessing remains both unavoidable and highly heterogeneous (Table 3 and Table 4). Specifically, 86.7% of the papers used between 1 and 4 of the 12 reported techniques, while 6.7% used no preprocessing at all. This pattern indicates a continuing lack of standardization, which hampers comparability. Segmentation (T03) and length normalization (T05) dominate the landscape, as seen in Figure 7, owing to the technical necessity of fixed-length inputs [155]. The fixed-window segmentation [61] is simpler to deal with than beat-based segmentation [84,91]. However, the first approach might not align with the clinical events of interest. The second approach will align with the clinical events and is more precise but it requires the use of manual feature engineering from the original signals, which is error-prone. This dichotomy indicates the trade-off between automation and clinician fidelity. The absence of a standard protocol is further reflected in amplitude normalization (T02), using Z-score mostly and Min–Max infrequently; this situation makes reproducibility difficult (D19). In the same way, noise and artifact removal (T01) is performed by digital bandpass filters having various cut-off frequencies, indicating different filtering criteria. Resampling (T04) introduces another source of variation because the sampling frequency is not agreed upon; it is commonly downsampled, resulting in a loss of resolution. Additional techniques, including the initial cleaning of data (T07) [75,110], are generally performed manually and considered optional. This highlights concerns about the robustness of the model under real-world inference. In the same way, the data balancing class (T06, T08) is hardly used, which indicates that there were no efforts taken to prevent the establishment of bias or to improve generalizability. Thus, while end-to-end DL models are oriented towards minimal and automatic preprocessing, it is nevertheless an essential component. It is even more important that the varied parameters and configurations used across studies diminish comparability and prevent meaningful conclusions. The evidence suggests that the field is still in flux: aiming for end-to-end automation but being foiled by the absence of standardized preprocessing pipelines.

The surveyed literature identified the aforementioned two vital difficulties in preprocessing that could influence the performance and generalizability of the models (Table 13). The first one refers to the high level of noise and artifacts, which were not taken into consideration during training (D43). Although this approach helps with this issue, misclassification still occurs in the presence of huge noise [78]. This indicates that clinically oriented models should include a separate noise class along with a normal and a pathological class to reject highly corrupted signals. The second problem concerns the unrealistic generation of the synthetic data (D44), leading to fake patterns [128]. When balancing techniques are used, synthetic data can reproduce biases from the original clinical datasets (such as population composition, acquisition protocols, or labeling practices) [170]. As a result, this decreases generalization or explainability. Beyond these challenges discussed, there are more limitations left unaddressed (Table 15). One of the most paramount challenges is the lack of automatic signal quality control in real life (D56), not only for excessive noise but also for loss of signal, saturation, and baseline drift. Another major problem is the lack of a common standard for mapping out the multichannel preprocessing across heterogeneous acquisition devices (D55). Interoperability problems and data approach issues limit models’ applicability in different clinical settings. The results of these findings, taken together, require the automated preprocessing strategies used to be adaptive and robust to a wide range of scenarios [74] and fully integrated in the pipeline for real-world use [18]. Preprocessing is essential; however, it continues to be diverse, subjective, and human-driven. These factors hamper reproducibility and limit the generalization of DL models to uncontrolled clinical scenarios.

### 7.2. About End-to-End DL Techniques

The wide variety of end-to-end DL models for ECG-based CAIC (Table 5) suggests a rapidly evolving field. Hybrid CNN-based techniques like CNN–BiLSTM and CNN–BiGRU, along with DenseNet, ShuffleNet, and SqueezeNet [20,21,83,85], continue to dominate with the dual purpose of capturing temporal information and spatial representation [84]. Despite this, there is a growing interest in newer architectures, namely, transformers and attention networks, which learn to model global dependencies and can scale better. Apart from the architecture, the increasing maturity of methodological innovations include contrastive learning [73], multitask and continuous learning [55], transfer learning, autoencoders, and knowledge distillation [68]. In summary, these approaches indicate a growing interest in autonomous and generalizable models as a way to tackle the issues of multi-derivation, multi-class, and multi-label classification. Emerging paradigms are suggesting that the convergence of hybrid CNNs is signaling a turning point in the field, with solutions based on systems rather than incremental improvements aimed at efficiency, adaptability, and clinical relevance.

Despite enhancements, end-to-end deep learning techniques continue to encounter significant obstacles for deployment in hospitals (Table 10, D14). The hurdle for the architectures (D02), which entails their complexity, poses the largest trouble and includes problems related to large annotated datasets, hardware requirements, and fine-tuning. This constraint limits portability (D08) and implementation in low-resource contexts. Utilizing several leads (D03) heightens the complexity of the model, which may result in increased overfitting risk when data are scarce or imbalanced [73]. Similarly, long-sequence (D01) analysis has yielded hybrid CNN designs that capture temporal dependencies of higher complexity. Biases in methodology still exist, and the lack of external cross-validation (D15) has a debilitating effect on robustness, as many models fail when their use is extended over different equipment, environments, or patients [18]. The issue is broader: end-to-end DL models are sensitive to the bias of the training data and perform worse on out-of-distribution and out-of-typical-distribution scenarios with comorbidities. Undefined challenges (Table 15) aggravate these constraints. Despite the existence of sophisticated optimizers [59], D58 hyperparameter optimization is still mostly manual. The use of semi-supervised learning (D59), like contrastive learning [40,73], to make better use of unlabeled data could be a great solution. Similarly, the limited diagnostic capacity of the algorithms is due to their lack of real-time adaptability (D54); continuous learning [55] can overcome this limitation and enable models to incorporate new expressions of cardiac diseases while retaining acquired knowledge. Overall, both the explicit and the overlooked challenges call for the need for efficient strategies and personalization mechanisms. End-to-end deep learning models require strengthening of validity, generalizability, and robustness in order to go from proof-of-concept to reliable tools for clinical use.

### 7.3. About Databases

**Figure 11:**
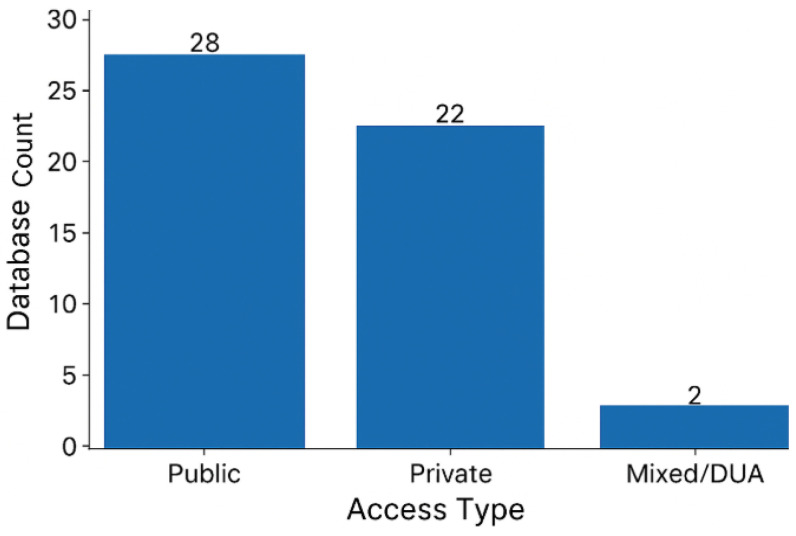
Public vs. private ECG data: a barrier to benchmarking?

**Figure 12:**
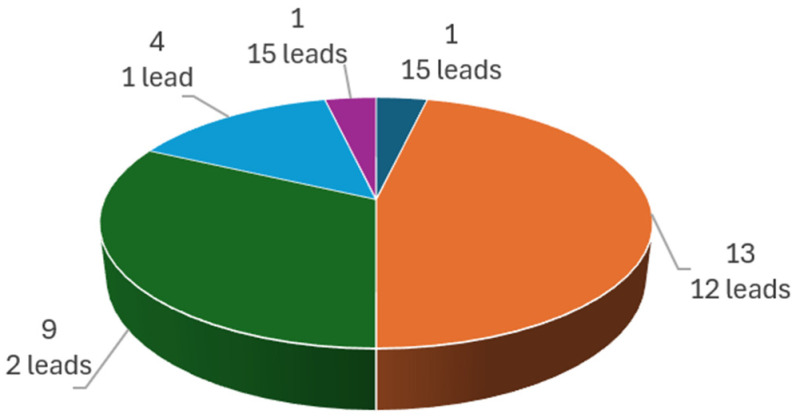
Lead diversity in public ECG datasets: a challenge for model generalization.

The inventory revealed the use of 52 different ECG databases (see Table 6 and Table 7) but with significant variability with respect to class definitions, sampling frequency, number of records, number of leads, duration of recording, and availability of datasets. The presence of this heterogeneity indicates a severe lack of standardization (D19) that undermines model transferability and comparability critically [171]. Only 17 databases were reused across studies (Table S3), while 35 appeared only once (Table S4). The reuse of such few resources signifies the cleft benchmarking practices witnessed across the NLG domain. Figure 8 also shows that seven databases—CPSC-2018, PTB-XL, MIT-BIH, AFDB, CinC2017, Chapman–Shaoxing, and MIT-BIH—together account for 82.6% of the usage. An excessive reliance on any single dataset, mainly for fine-tuning a model, can incur domain bias and restrict inter-dataset generalization (D23) [16]. While a model may perform convincingly on a single source, it may not necessarily extend its range of efficiency to other sources [29]. These worries are echoed in Figure 11 and Figure 12. As illustrated in Figure 11, nearly half of the databases, out of the 52, are private. Furthermore, the other 28 are solely publicly accessible. Finally, two come under restricted Data Use Agreements. According to Table 6, 46.15% of databases have no public access (D21), obstructing transparency, reproducibility, and collaborative advancement. Of the public datasets, only 13 contain 12-lead recordings, the distribution of lead diversity is illustrated in Figure 12. Others offer far fewer leads, such as one, two, or fifteen. This makes it difficult to generalize our model to other acquisition setups. The majority of the 31 databases, supporting 12 derivations and 16 multi-labels (most of which also use 12 leads), have similar technical characteristics. However, the technical richness is not evenly distributed and is often restricted to the most reused datasets. Consequently, a relative wealth of resources is available but researchers tend to use an unrepresentative subset due to access limits and benchmarking bias. In order to address the above limitations, it has been argued that future research should use a multiplicity of data and report results over databases not used for the training [59,71]. This would make a broader validation of the results possible and reduce the risk of becoming too specific to the dataset.

The studies in Table 11 that discuss the reported issues reveal major shortcomings that clearly affect robustness, generalizability, and clinical relevance. Class imbalance (D17) is one of the issues that biases the model in favor of the majority class, with performance losses for all other clinically important conditions. Another common issue is the lack of large, diverse, and well-annotated datasets (D18), which poses challenges for adequate training. Besides these stated impediments, there is an unraised challenge: the development of databases with synthetic signals (D61) to introduce difficult-to-capture rare or paroxysmal patterns resembling real signals in order to incorporate events of rare occurrence [88]. The task overlaps with D44 (unrealistic synthetic data generation), where the model may learn the non-existent and, thus, result in a lack of generalizability and explainability [85]. As such, synthetic data should be sufficiently faithful to the complex form and change patterns in varied pathology (D40, D41). In this paper’s subsection, we will expand on the challenge posed by D61, as well as the significance of overcoming it in allowing end-to-end DL techniques to lessen the dependence on costly expert annotation (D18) and improve class equity by better representing minority classes. All these challenges, taken together, indicate that databases are not just a collection of data [84], but rather they are the cause of several serious limitations; databases should not just be accessible but also diverse, standardized, and clinically supported in order to enable building robust high-performing models in common clinical settings [172].

### 7.4. About Cardiac Pathologies: Cardiac Arrhythmias and Ischemia

The cardiac pathologies inventory (Tables S3 and S4) suggests that the studies tackled a very high number of pathologies, i.e., 153 pathologies (not merely conditions). However, the said data are inequitably represented in the studies, exhibiting a bias in focus. According to Figure 9, only a handful of cardiac conditions have been mainly researched since 69% of studies covered atrial fibrillation. Further, the other major studies also involve PVC and PAC. Diseases that are rarer or more complex than others receive less attention. This trend illustrates the long-tail issue [107,173], which constrains their clinical applicability and diagnostic value for the variety of diseases less often covered in research; this is also true for other models.

Cardiac conditions pose intrinsic challenges (Table 12) due to their dynamic physiology and definition in ECG expression (D40) [55], which often leads to misclassification. The subtle differences between arrhythmias such as AF and AFL (D34) create complications for multi-class tasks, while high intra- and inter-patient variability (D36) impedes generalization. It is observed clinically that comorbidities (D35) are quite common; however, this area is poorly studied [71,113,174]. Episodic conditions such as paroxysmal AF [153] need long recordings (such as Holter and patches) [92,110]. On the other hand, persistent rhythm scenarios were focused on models. One critical gap is neglecting the former expression for dynamic pathology changes (D62). Existing models use fixed windows [101], failing to account for the evolution of other disease like infarction progression or AF transition. The utility in a clinical context is, therefore, reliant on classifications made at a given moment in time as well as on tracking the evolution through time [110]. In conclusion, these limitations thus advocate for the need to build more adaptive and robust DL models to cope with the variability and complexity of cardiac diseases.

### 7.5. About Evaluation Metrics

**Figure 13:**
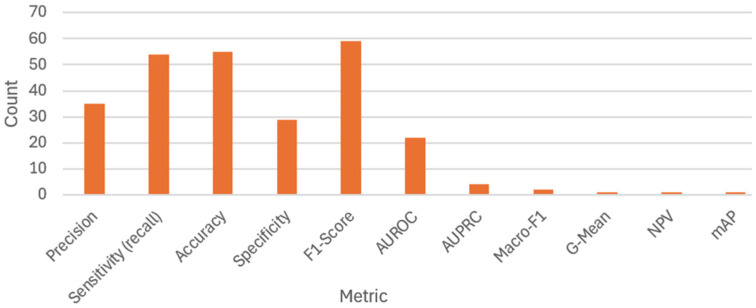
Frequency of performance metrics used in the selected studies.

The metrics used in the studies reviewed, both per class and aggregated, included 11 different metrics (Table 7). The three most commonly used metrics were recall, accuracy, and F1-score. The F1-score is the harmonic mean of precision (M01) and sensitivity (M02), and is especially useful for unbalanced datasets [94]. However, accuracy, by itself, is a misleading metric since it may be inflated by true negatives or dominating classes like normal rhythm. Figure 13 establishes the trend. The usage of F1-score, recall, and accuracy by far dominates in usage. As for the specialized metrics AUPRC, NPV, G-Mean, and mAP, they are rarely applied. AUROC, specificity, and precision exhibited an average value of (about) 95 percent with low dispersion, but they are still biased toward describing the majority class performance. It is worth noting that only four studies [76,175] made use of AUPRC, which is the more useful metric for rare events, whereas Macro-F1 (M08) and mAP (M11) occurred in just two and one studies, respectively. The performance of minority classes is often underreported to mask the weaknesses of the clinical applicability of the method. Using global metrics because they are widely accepted instead of the more class-conscious ones indicates a benchmarking bias that undermines evaluations of long-tail pathologies. To improve robustness and fairness, future studies should aim to use metrics that look at performance across all classes, particularly in uneven and multi-label situations.

While traditional metrics are still commonly used, there is no standardization in their use (D45). This causes a problem as comparing studies becomes impossible. One of the unaddressed issues (see Table 13) concerns the low usage of metrics that evaluate either explanation quality or confidence (D63), which is important for the clinical uptake [172]. In addition, the metric selected is often driven by statistical convenience rather than medical relevance (D64), and the potential impact of a false negative is often overlooked; in the case of arrhythmia, this could be a delayed diagnosis. Accuracy, for example, does not reflect these clinical consequences [107]. The described failings relate to problems of explainability, such as the inability to retrace reasoning (D51), the lack of validation of highlighted regions (D53), or uncertainty that visualizations (e.g., Grad-CAM) are consistent with cardiological knowledge (D49). Without solid evaluation, we do not trust the “black box” (D02). In summary, the data suggest that a standardized evaluation framework aligned with clinical goals is necessary to validate models technically and medically.

### 7.6. About Explainability Techniques

**Figure 14:**
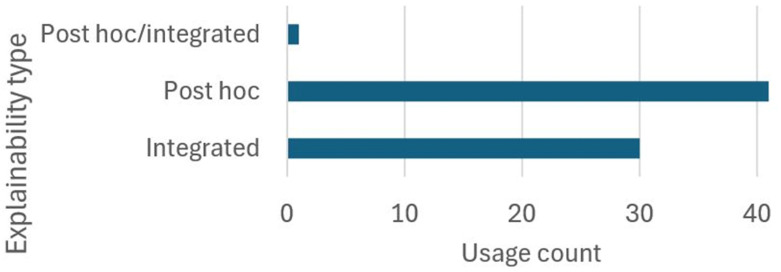
Distribution of explainability types in reviewed studies.

The clinical adoption of DL models is hampered due to limited model explainability [76,78]. Of the 121 studies reviewed, only 59 of the studies used at least 1 of the 23 techniques (Table 8), indicating low priority. There are two kinds of interpretability techniques: post hoc (like Grad-CAM, salience maps, SHAP, and t-SNE) and integrated (like attention and NBET). As shown in Figure 14, post hoc methods are the most used methods among interpreters. Both techniques Grad-CAM and t-SNE are the most used post hoc techniques, whereby Grad-CAM was the technique most commonly encountered in 18 studies as a visualization tool for indicating important ECG regions. Out of the 18 studies analyzed, attention mechanisms are the most common integrated approach [108] for improved training transparency. Overall, the real impact of integrated means (especially spatial and temporal attention (TE02 and TE08)) is not effective explainability. The limited use of hybrid or post hoc/integrated approaches indicates a failed attempt to align interpretability with complexity. The evidence indicates that explainability is viewed as important, but implementation is sporadic and often superficial, limiting trust and clinical uptake.

In spite of certain accomplishments, the clinical utility of current capabilities is limited by eight reported shortcomings. Attention mechanisms in ECG signal processing may fixate on unrelated areas (D46), while the Grad-CAM maps may share too much overlap (D48), thus leaving attributing the model prediction open to interpretation. The clinical applicability is further constrained by five more challenges (Table 15) that have not been addressed. Customized visual tools are lacking with respect to long-term recordings such as Holter data (D67). This makes it difficult to find episodic or late-onset events (D71). The visual techniques also face challenges in a multi-label or multi-lead context (D69). This is because merged explanations do not indicate which class or lead contributes to which output (D68). For instance, myocardial infarction (MI) relies on careful analysis of the ST segment and the T wave, while atrial fibrillation (AF) relies on the rhythm and the absence of any P waves [107]; without validation specific to pathology, explainability will lose its clinical relevance. Another significant disparity is the unavailability of reference metrics to gauge concordance with clinical expectations (D70). Without tools for response evaluation (D63), we cannot quantify how much we may trust a model. Consequently, these limitations show that current techniques may improve transparency, but do not yet suffice for deployment in the clinic [176]. There is an urgent need for temporally sensitive explainability methods, pathology-adapted and compatible with long-term multi-channel recordings for reliable automated diagnosis [177].

### 7.7. Limitations

This study has some limitations. First, the analysis focused exclusively on six key aspects of CAIC with ECG and end-to-end DL, neglecting other relevant dimensions such as ethical and regulatory issues, implementation in portable or embedded hardware, and integration into clinical settings. Clinical validation of this study was not conducted; thus, this study may be limited from a medical point of view. In the end, although the new challenges identified in this study did not appear in the articles we reviewed, the need to validate them remains an important task for the near future to decide if they may hamper the development of robust and clinically useful DL models.

## 8. Conclusions

The aim of this review was to investigate CAIC with ECG and end-to-end DL techniques. This article examined the key challenges associated with them. The challenges are in the aspects of preprocessing, DL techniques, databases, cardiac pathologies, evaluation metrics, and explainability techniques. The results include extensive inventories for these six areas based on relevant, impactful studies, as well as technical barriers that limit CAIC performance and clinical implementation. Collectively, this provides a systematic overview of the current state of the field. Unlike other CAIC with DL and ECG reviews, this study only focused on end-to-end DL, where 71 challenges were identified, which are as follows: 53 found in the literature, and 18 that are still not addressed. We should consider these latter challenges to close the gap toward high-performing models. This paper indicates that preprocessing conducted at the end-to-end level of DL models is minimal, transparent, and automated to improve performance while adding no unnecessary complexity. Despite the encouraging results obtained using these architectures, the issues of generalizability and training complexity persist. In addition, there are increasing calls for databases that are more diverse and better balanced between classes, particularly for pathologies with similar morphologies, such as AF and AFL, that make the problem more complicated. There is a need for performance evaluation metrics aligned with clinical practice, as well as for more robust and explainable techniques applicable to a wider range of clinical situations. This study offers a firm basis for designing more generalizable, robust, and clinically useful solutions.

As future work, we propose conducting studies to address the identified difficulties and to accelerate the advancement of CAIC through ECG and end-to-end DL. Additionally, we recommend creating a comprehensive framework for restoring and maintaining normal heart rhythm through the classification, prediction, explanation, treatment, and simulation of arrhythmias and cardiac ischemia—an approach similar to that employed by [178]—to maximize survival in pediatric congenital heart surgery.

## Figures and Tables

**Table 1:** Inclusion and exclusion criteria.

Inclusion Criteria	Exclusion Criteria
Addresses the research question Study Type: Original journal article Language: English Period: 2019 to 2025	Studies not aligned with the objectives of this review. Studies conducted in different contexts (e.g., sleep disorders, diabetes, neonates or fetuses, non-human subjects, drug effects, recent surgeries). Studies focusing on other aspects (e.g., risk factors, treatments, prevention, use of tools other than ECG such as radar, echocardiography, or pulse oximetry, or not employing end-to-end DL). Conference proceedings, posters, editorials, and theses. Studies without contributions or results.

**Table 2:** Potentially eligible and selected articles.

Source	Potentially Eligible Articles (*n*)	Selected Articles (*n*)	Selected Articles
Scopus	478	35	[19,21,40,53,54,55,56,57,58,59,60,61,62,63,64,65,66,67,68,69,70,71,72]
Web of Science	1091	68	[17,18,20,73,74,75,76,77,78,79,80,81,82,83,84,85,86,87,88,89,90,91,92,93,94,95,96,97,98,99,100,101,102,103,104,105,106,107,108,109,110,111,112,113,114,115,116,117,118,119,120,121,122,123,124,125,126,127,128,129,130,131,132,133,134,135,136]
PubMed	0	0	---
IEEE Xplore	447	18	[137,138,139,140,141,142,143,144,145,146,147,148,149,150,151,152,153,154]
Total	2016	121	[13,14,15,16,17,30,41,42,43,44,45,46,47,48,49,50,51,32,52,53,54,55,56,57,58,59,60,61,62,63,64,65,66,67,68,69,70,71,72,73,74,75,76,77,78,79,80,81,82,83,84,85,86,87,88,89,90,91,92,93,94,95,96,97,98,99,100,101,102,103,104,105,106,107,108,109,110,111,112,113,114,115,116,117,118,119,120,121,122,123]

**Table 3:** Types and descriptions of preprocessing techniques.

ID	Technique Type	Description	Usage Count	References
T01	Noise and Artifact Removal	*Noise*: Unwanted random signals with a broad spectrum and low amplitude (~0.01–0.1 mV) superimposed on the ECG signal, including thermal and electronic noise. *Artifacts*: Higher-amplitude distortions (~0.1–10 mV) caused by physiological factors (breathing, muscle movement, physical activity, sweating, pacemakers), technical issues (poor electrode contact, faulty cables), or environmental factors (vibrations, 50/60 Hz interference). Their spectral range is ~0.05–100 Hz and they appear as abrupt spikes, irregular waves, interruptions, or baseline fluctuations around 0.05 Hz.	61	[17,19,20,21,40,55,60,64,65,71,72,73,76,79,81,83,87,88,90,94,96,98,99,104,105,106,108,110,112,114,115,119,120,121,122,124,126,130,132,135,136,137,141,142,143,145,146,153,156,157,158,159,160,161,162,163,164,165,166,167]
T02	Amplitude Normalization	Scaling ECG amplitudes into the same range to improve comparability and reduce scale bias.	57	[17,19,21,55,60,61,62,63,65,66,68,71,74,75,78,79,80,84,85,86,87,88,90,91,92,98,99,102,104,106,110,115,120,121,122,123,124,127,129,131,132,135,137,141,149,151,153,154,156,159,162,163,164,165,166,167,168]
T03	Segmentation	Dividing the signal into fixed-length segments for efficient processing because DL models require fixed-length inputs.	66	[17,18,19,21,40,55,64,65,66,75,78,79,80,83,85,86,87,88,89,90,91,93,95,96,97,99,102,104,105,106,107,108,110,113,114,115,117,118,120,121,122,124,125,126,128,132,133,138,141,142,149,151,153,154,156,157,158,159,160,161,162,163,164,169]
T04	Resampling	Ensuring consistent sampling frequencies when using multiple databases. Downsampling is often used to reduce computational load.	42	[17,18,19,21,40,53,58,65,66,68,78,87,91,96,99,100,102,104,108,110,113,115,116,121,122,123,124,125,131,132,134,149,151,153,154,156,158,159,162,163,164]
T05	Length Normalization	Applying techniques like padding and cropping to equalize signal length across ECG records. Required because DL models need fixed-length inputs.	30	[18,40,58,60,61,63,67,68,70,71,78,84,85,88,89,91,93,95,101,103,112,116,127,129,131,134,141,151,162,163]
T06	Class Balancing	Adjusting class distribution in datasets when classes are unevenly represented.	16	[20,63,64,75,80,87,92,93,106,107,114,118,133,144,146,153]
T07	Data Cleaning	Correcting or removing missing, duplicate, or invalid data, including the removal of noisy sections (clipping).	13	[20,67,75,76,80,82,83,85,97,98,110,114,140]
T08	Data Augmentation	Enhancing model robustness through synthetic data generation or transformations. May also help balance class distribution.	16	[21,53,58,59,65,85,103,110,112,128,129,140,143,145,149,167]
T09	Z-shaped Reconstruction	Converting one-dimensional data into two-dimensional representations.	1	[105]
T10	Lead Expansion	Creating new leads by mathematically combining existing ones.	2	[67,68]
T11	Wavelet Decomposition	Decomposing the ECG signal into different frequencies or scales to extract features at each level.	2	[80,109]
T12	Inter-Patient Variability Reduction	Minimizing ECG variability across patients with the same pathology to improve the generalization of DL models.	1	[135]

**Table 4:** Preprocessing specific techniques.

ID	Technique Type	Specific Techniques	Usage Count
T01	Noise and Artifact Removal	Wavelet	8
		Digital filter	21
		LOESS	1
		Moving average	1
		Smoothing	1
		NLM	2
		Normalization	1
		Thresholding	2
T02	Amplitude Normalization	Z-score	49
		Min–Max	7
		Unit variance	1
T03	Segmentation	Fixed window	60
		Multiple fixed windows	3
		Overlapping sliding windows	3
T04	Resampling	Downsampling	40
		Upsampling	2
T05	Length Normalization	Zero-padding	11
		Cropping	11
		Trimming	5
		Replication	2
		Segmentation	1
		Resampling	3
		Filling	4
T06	Class Balancing	Oversampling: SMOTE	4
		Oversampling: GAN	1
		Oversampling: ADYSAN	1
		Downsampling	2
		Oversampling	1
		Replication	2
		Segmentation	2
		Data amplification	2
T07	Data Cleaning	Remove missing values	2
		Remove zeros or NaN data	2
		Remove noisy segments	5
		Remove duplicates	1
		Remove anomalous portions	2
T08	Data Augmentation	Cropping	1
		Jittering	1
		Warping	1
		Noise injection	2
		Scaling	2
		Random sampling	2
		Others	5
T09	Z-shaped Reconstruction	---	1
T10	Lead Expansion	---	2
T11	Wavelet Decomposition	---	2
T12	Inter-Patient Variability Reduction	FFT- and Hanning window-based filter	1

**Table 5:** End-to-end DL technique families.

Family	Representative Techniques	Usage Count	References
CNN-based models	CNN, ResNet, DenseNet, Inception, SE-ResNet, ShuffleNet, U-Net, AlexNet-1D, Multi-Resolution CNN, Temporal/Dilated CNN, GoogLeNet, XResNet	35	[55,57,62,73,76,77,78,80,86,87,89,91,95,97,98,100,102,106,109,114,118,119,128,133,136,140,144,148,149,152,154,156,169]
RNN-based models	LSTM, Bi-LSTM, GRU, BiGRU, Elman	5	[64,74,81,82,94]
Hybrid CNN-RNN models	CNN–LSTM, CNN–BiLSTM, CNN–GRU, CNN–BiGRU, CNN–BiLSTM–BiGRU, Deep CNN–LSTM	23	[17,19,63,84,92,93,99,112,120,123,124,125,126,130,132,135,139,142,143,161,164,167]
Transformer-based models	CNN–Transformer, Swin–Transformer, Dual-view Transformers	8	[21,54,83,108,131,145,159,166]
Attention-enhanced models	SE blocks, channel/spatial/temporal attention, multi-head attention, CNN + SSM	39	[18,19,56,59,60,61,65,67,68,69,70,71,79,85,88,90,101,103,104,105,113,115,116,117,121,122,129,134,137,138,141,146,150,151,153,157,158,160,165]
Generative/Contrastive	Autoencoder, Contrastive Learning	7	[40,53,58,96,110,162,163]
Custom/Ensemble/Neural Architecture Search	Reinforcement Learning, Bat Algorithm, Binarized Neural Network, AlexNet-1D Semi-supervised	4	[66,111,127,168]

**Table 6:** Characteristics of the databases used.

ID	Database	fs (Hz)	No. of Records	Record Duration	Access	Leads Used
DB01 ^a^	AHA ECG Database (AHA)	500	45,152	10 s	PUB	12
DB02	Asan Medical Center Liver Transplant Database	500	65,932	10 s	PRIV	12
DB03 ^a^	AUMC ICU Biosignal Database	500	190,000	10–20 s	PRIV	12
DB04 ^a^	Author-collected dataset	500	6877	6–60 s	PUB	12
DB05	Chinese PLA General Hospital	200	1436	6 s–30 min	PUB	I, II
DB06	CPSC-2018 (public set + CPSC-Extra)	250	35	8 min	PUB	1
DB07	CPSC-2020	250	35	2 h	PUB	2
DB08 ^a^	CPSC-2021 (V1.0.3)	500	10,344	5–10 s	PUB	12
DB09 ^a^	Custom wearable ECG device recordings	500	32,142	10 s	PRIV	I, II, V1–V6
DB10	Datasets from South Korean University Hospitals	257	75	30 min	PUB	12
DB11	ECG Arrhythmia Classification Dataset	360	48	30 min	PUB	2
DB12	Federal Ministry of Education and Research Dataset	250	25	10 h	PUB	2
DB13	First Affiliated Hospital of Nanjing Medical University ECG Database	300	8528	9–61 s	PUB	1
DB14	First People’s Hospital of Guangzhou Database	1000	549	2 min	PUB	12, 3 Frank
DB15 ^a^	Korea University Anam Hospital ECG Dataset	100, 500	21,799	10 s	PUB	12
DB16	Lobachevsky University Database (LUDB)	500	200	10 s	PUB	12
DB17	Long-Term AF Database (LTAFDB)	500	45,152	10 s	PUB	12
DB18	Mayo Clinic ECG Database	250	80	3 h	PRIV	2
DB19	MIMIC-III	125	436	NR	PRIV	II
DB20	MIT-BIH Malignant Ventricular Arrhythmia Database (VFDB)	250, 500	2,648,100	NR	PRIV	II
DB21	MIT-BIH Noise Stress Test Database (NSTDB)	NR	6500	NR	PRIV	12
DB22	MIT-BIH Supraventricular Arrhythmia Database (SVDB)	500	NR	10 s	PRIV	12
DB23 ^a^	Patch Database	500	13,256	6–144 s	PUB/PRIV	12
DB24 ^a^	PhysioNet 2020	400	10	~24 h	PUB	1
DB25 ^a^	Private 12-lead ECG Dataset	100–1000	>100,000	5 s–30 min	PUB	12
DB26	QT Database (QTDB)	400	29	24 h	PRIV	I
DB27 ^a^	Shandong Provincial Hospital Database (SPHw) ^a^	NR	52,043	10 s	PRIV	II
DB28	Shandong Provincial Hospital Database (SPH)	NR	5000	10 s	PRIV	12
DB29	Shandong Provincial Hospital Database (SPHDB) ^a^	512	16,000	120 s	PRIV	I, II
DB30	Shanghai Ninth People’s Hospital Database (SNPH)	NR	277,807	10–60 s	PRIV	12
DB31	Shanxi Bethune Hospital Dataset	1000	90	10 s	PRIV	12
DB32	Telehealth Network Minas Gerais (TNMG)	200	28,308	10 s	PUB	II
DB33 ^a^	Third Affiliated Hospital of Sun Yat-sen University Database	500	200	10 s	PUB	12
DB34	Wearable ECG device recordings	128	84	24–25 h	PUB	2
DB35 ^a^	Wearable long-term ECG device recordings	500	2,499,522	~10 s	DUA	12
DB36	AHA ECG Database (AHA)	125	>67,000	Up to several weeks	PUB	I, II, III, aVR, V
DB37	Asan Medical Center Liver Transplant Database	250	22	30 min	PUB	2
DB38	AUMC ICU Biosignal Database	360	12	30 min	PUB	2
DB39	Author-collected dataset	128	78	30 min	PUB	2
DB40	Chinese PLA General Hospital	NR	328	30 s	PRIV	1
DB41 ^a^	CPSC-2018 (public set + CPSC-Extra)	100–1000	43,101	5 s–30 min	PUB	12
DB42	CPSC-2020	NR	549,211	NR	PRIV	12
DB43	CPSC-2021 (V1.0.3)	250	105	15 m	PUB	2
DB44	Custom wearable ECG device recordings	200	NR	24 h	PUB	12
DB45 ^a^	Datasets from South Korean University Hospitals	500	25,770	10–60 s	PUB	12
DB46	ECG Arrhythmia Classification Dataset	200	NR	24 h	PUB	12
DB47	Federal Ministry of Education and Research Dataset	500	75,111	11–92 s	PRIV	12
DB48 ^a^	First Affiliated Hospital of Nanjing Medical University ECG Database	500	7000	NR	PRIV	12
DB49 ^a^	First People’s Hospital of Guangzhou Database	300–600	2,322,513	7–10 s	PUB	12
DB50	Korea University Anam Hospital ECG Dataset	1000	793	10 s	PRIV	12
DB51	Lobachevsky University Database (LUDB)	500	5189	NR	PRIV	12
DB52	Long-Term AF Database (LTAFDB)	400	12	~2 days	PRIV	12

NR: not reported by the authors, ^a^ multi-label database, PUB: public, PRIV: private, DUA: Data Use Agreement.

**Table 7:** Metrics used.

ID	Metric	Count	References
M01	Precision	35	[17,21,55,57,61,62,63,64,66,71,76,77,82,83,84,87,88,89,91,93,95,98,99,102,104,111,112,113,120,121,122,128,130,133,134]
M02	Sensitivity (recall)	54	[17,20,21,53,55,56,57,61,62,63,64,66,67,68,70,71,73,76,77,80,81,82,83,84,87,88,89,91,92,93,94,95,98,99,102,103,104,108,110,111,112,113,118,120,122,123,125,126,127,128,129,132,133,135]
M03	Accuracy	55	[17,18,20,21,54,55,56,57,58,61,62,63,65,66,67,68,69,71,77,78,80,81,83,85,86,87,88,89,90,91,92,94,95,97,99,102,103,104,107,108,110,111,112,113,115,116,117,118,125,126,127,128,131,134,136]
M04	Specificity	29	[21,56,62,66,67,71,73,76,80,81,82,87,89,92,94,102,103,104,110,111,112,123,125,126,127,129,132,134,135]
M05	F1-Score	59	[17,18,20,21,40,54,55,56,57,60,61,62,63,64,69,70,74,75,76,77,79,81,82,83,84,86,87,88,89,91,93,95,97,98,99,100,101,104,105,106,108,110,111,112,114,115,116,117,118,120,121,122,129,130,131,132,133,134,136]
M06	AUROC	22	[18,53,57,59,60,61,62,68,70,75,76,85,90,98,99,109,115,116,117,123,129,132]
M07	AUPRC	4	[18,59,85,117]
M08	Macro-F1	2	[90,128]
M09	G-Mean	1	[112]
M10	NPV	1	[129]
M11	mAP	1	[70]

**Table 8:** Explainability techniques used.

ID	Technique	Description	Type of Explainability	Studies
TE01	Activation Maps	Enable understanding of how a model processes inputs across different convolutional layers.	Post hoc Integrated	[118]
TE02	Attention Maps	Visualize the spatial or temporal distribution of the attention learned by the model.	Post hoc	[115]
TE03	Attention Mechanism	Allocates weights to portions of the input according to their importance for classification.	Integrated	[18,21,54,56,59,61,67,85,88,103,104,113,116,117,123,129,131,134,138,141,146,157,159,165,166]
TE04	Embedding Visualization	Shows how internal representations are organized in the model’s latent space.	Post hoc	[83]
TE05	Feature Heatmaps	Highlight the local importance of features over the input, typically in the temporal domain.	Post hoc	[105]
TE06	Global Channel Attention Block (GCAB)	Assigns weights to input channels to emphasize the most relevant ones.	Integrated	[101]
TE07	Grad-CAM	Generates activation maps to identify the input regions most relevant for prediction.	Post hoc	[21,60,65,68,84,91,93,102,131,134,137,145,154,156,160,161,166,168]
TE08	Gradient-based Visualization	Visualizes gradient magnitudes with respect to the input as an indicator of importance.	Post hoc	[121]
TE09	Heatmaps	Display the intensity of a feature at each point of input.	Post hoc	[114]
TE10	Integrated Gradients	Accumulates gradients between a baseline signal and the actual input to estimate feature importance.	Post hoc	[78]
TE11	Layer-wise Relevance Propagation (LRP)	Propagates relevance scores back to the input layers to identify key regions.	Post hoc	[78]
TE12	Lead-wise Grad-CAM	Applies Grad-CAM individually to each ECG lead to show its contribution to the prediction.	Post hoc	[103]
TE13	Neural-Backed Ensemble Trees (NBET)	Combines decision trees with neural networks to improve model interpretability.	Integrated	[97,98]
TE14	Optimal Energy Classifier	Applies a minimum-energy principle to identify discriminative features.	Integrated	[99]
TE15	Principal Component Analysis (PCA)	Reduces the dimensionality of representations for visual analysis or pattern identification.	Post hoc	[79]
TE16	SHapley Additive exPlanations (SHAP)	Evaluates the contribution of each feature using principles from game theory.	Post hoc	[76,158]
TE17	Saliency Mapping	Creates sensitivity maps that highlight the influence of each feature on the results.	Post hoc	[79,99]
TE18	Self-attention	Assesses relationships between input elements with respect to themselves to assign relevance.	Integrated	[20]
TE19	Semantic Transformations	Apply semantic transformations to evaluate the model’s robustness and its understanding.	Post hoc	[58]
TE20	Sensitivity Maps	Indicate how the model’s output varies when parts of the input are modified.	Post hoc	[97]
TE21	Similarity Matrix of Embeddings	Represents the similarity between embeddings to understand learned internal relationships.	Post hoc	[83]
TE22	t-Distributed Stochastic Neighbor Embedding (t-SNE)	Permits analysis and visualization of high-dimensional data, often in combination with other techniques to enhance representations.	Post hoc	[66,76,79,105,108,153]
TE23	Weight-based Recursive Feature Elimination	Iteratively removes the least relevant features based on the learned weights.	Post hoc	[72]

**Table 9:** Purpose of the aspects of difficulty analysis.

ID	Aspect of Difficulty Analysis	Purpose
AA1	Preprocessing	Enhance data quality and usability so models can learn and infer with greater efficiency and accuracy.
AA2	End-to-end DL techniques	Allow the model to automatically learn relevant features and classify arrhythmias and ischemia directly from data, without manual feature extraction or expert intervention.
AA3	Database	Supply high-quality, well-labeled, and balanced data with demographic and pathological diversity of sufficient size and with clear documentation to support accurate ECG-based classification of cardiac pathologies.
AA4	Cardiac pathologies	Serve as diagnostic targets for the model, providing the classes it must learn to classify from ECG signals.
AA5	Metrics	Provide objective and quantitative measures of model performance in classifying cardiac pathologies and excluding non-pathological cases.
AA6	Explainability techniques	Clarify and provide support for the model’s results.

**Table 10:** Difficulties in end-to-end DL techniques.

ID	Difficulty	Effects	References
D01	Long sequences	Increase resource consumption and cause processing latency, as well as temporal and clinical bias for transient events.	[93]
D02	Model complexity (black box)	Requires large amounts of data, computational resources, and careful tuning. Expensive to train. Limited use on portable or real-time devices. Reduces explainability.	[17,54,59,60,62,76,77,78,80,82,83,86,89,91,92,95,99,102,119,131]
D03	Use of multiple leads	Raises model complexity, computational load, and resource demand. Requires coherent integration of signals and more labeled data. Unsuitable for portable devices.	[56,58,62,68,80,82,95,101,103,107,128,134]
D04	High memory and CPU/GPU requirements	Lead to higher energy consumption and prevent implementation in real time or on resource-constrained hardware.	[54,55,79,91,118]
D05	Multi-labeling	Add complexity in design, training, and validation, and increase the difficulty of managing the output space.	[58,67,82,91,116]
D06	Large number of classes	Increases model complexity and computational demand while reducing accuracy for rare classes.	[21]
D07	Hyperparameter selection	Strongly affects performance and complicate adaptation to new domains or tasks.	[74,91,93,131]
D08	Embedded hardware or FPGA	Requires model optimization that may reduce performance. Involves high development complexity (FPGA) and limited resources in portable systems.	[17,20,79,80,82,99,103,109,113,118,127,128,131,134]
D09	Lack of standardization in architectures and protocols	Hinders comparison across studies, limits reproducibility, and complicates benchmarking, cross-validation, transfer, and clinical adoption.	[18,128]
D10	Scaling the model to other pathologies	Increases complexity, demands additional databases, hardware, and metrics, and further complicates explainability.	[17,82,83,121,123]
D11	Conversion to 2D spectrogram	Causes loss of fine morphological and temporal information, raises computational complexity, and reduces clinical interpretability.	[95]
D12	Personalization of the model for individual patients	Leads to overfitting due to limited patient data and failure from intra-individual variability.	[127]
D13	Dependence on large volumes of high-quality labeled data	Creates latency, high computational requirements, imbalance issues, and challenges in multimodal integration.	[20,62,107,121,133]
D14	Deployment in diverse real-world or clinical settings	Produces bias toward training datasets and poor performance under atypical conditions or comorbidities.	[20,67,69,72,82,83,85,90,95,100,108,123,125,126,127,128,129,131]
D15	External cross-validation	Omitting it inflates performance estimates and limits clinical acceptance.	[21]

## Data Availability

No new data were created or analyzed in this study. Data sharing is not applicable to this article.

## Supplementary Materials

The following supporting information can be downloaded at https://www.mdpi.com/article/10.3390/diagnostics16010161/s1, Table S1: End-to-end DL techniques; Table S2: Abbreviations of cardiac pathologies detected by ECG; Table S3: Cardiac pathology databases used in more than one study; Table S4: Cardiac pathology databases used in a single study; Table S5: Links to public databases; Table S6: The 14 most studied cardiac pathologies used; Table S7: Noise and artifact removal—specific techniques; Table S8: Preprocessing techniques T02–T05; Table S9: Preprocessing techniques T06–T12. Table S10. PRISMA 2020 for Abstract Checklist. Table S11: PRISMA 2020 Checklist.
