# Supplementary material for: Challenges in the Classification of Cardiac Arrhythmias and Ischemia Using End-to-End Deep Learning and the Electrocardiogram: A Systematic Review
Source: Diagnostics (Basel). 2026 Jan 4;16(1):161. doi: 10.3390/diagnostics16010161 (PMC12785992; doi:10.3390/diagnostics16010161)
Supplement: Supplementary file 1 [file diagnostics-16-00161-s001.zip › diagnostics-4026138-supplementary.pdf]

Supplementary Materials

Table S1. End-to-end DL techniques.

Author	Technique	Author	Technique
[1]	CNN–GRU with Farmland Fertility Algorithm	[2]	Multi-Scale CNN with SE
[3]	Multi-Scale 1D CNN	[4]	DenseNet-37
[5]	Spatio-temporal CNN with Global Average Pooling 5 (GAP 5)	[6]	CNN–BiLSTM
[7–11]	CNN	[12]	ResNet–BiLSTM
[13]	Pre-trained ResNet	[14]	ResNet with Attention Mechanism
[15]	1D Densely Connected Neural Network with Squeeze-and-Excitation (SE) and GAP	[16]	Convolutional Autoencoder with Transfer Learning (TL) and GoogLeNet backbone
[17]	Residual CNN enhanced with Multi-Scale Decomposition	[18]	Group Bi-LSTM and Residual Group CNN with Attention Mechanism and Multi-Task Learning
[19–21]	Bi-LSTM	[22]	Residual Block Ensemble Tree
[23]	CNN–Transformer with Multi-Head Attention	[24,25]	CNN with Residual Blocks
[26]	ResNet	[27]	CNN with ResNet Bottleneck Blocks
[28]	Contrastive Learning for ECG with 1D XResNet101 classifier	[29]	Recurrent CNN with Optimal Energy Classifier
[30]	CNN–BiLSTM Dynamic Graph Convolutional Network	[31]	1D CNN with Global Channel Attention and Short Residual Blocks
[32]	ResNet-18–BiGRU with Attention and SE	[33]	Visual-DenseNet
[34]	CNN–BiLSTM–BiGRU with Multi-Head Self-Attention	[35]	1D SE-ResNet with Lead-wise Attention Module
[36]	ResNet Variant with GAP	[37]	XResNet1D-50 with Supervised Contrastive Learning and Semantic Transformations
[38]	Modified CNN with Deformable Vision Transformer (Deformable ViT)	[39]	CNN–Depthwise Convolution with SE Cross-Guidance and Attention Fusion
[40]	CNN–Improved Bidirectional LSTM (IBLSTM)	[41]	CNN with Self-Attention Mechanism and GAN
[42]	Residual-based Temporal Attention CNN	[43]	Inception–GRU with Attention Module
[44]	Multi-Resolution Continual Learning CNN	[45]	Dual-view CNN–Transformers with External Attention Mechanism
[46]	ResNet34 with Multi-Label Correlation	[47]	Self-Complementary Attentional CNN

Table S1. End-to-end DL techniques (continued).

Author	Technique	Author	Technique
[48]	Improved Deep Residual CNN	[49]	CNN–BiLSTM–BiGRU with Efficient Channel Attention
[50]	CNN–GAN	[51]	CNN–Modified Bi-LSTM with Attention Mechanism
[52]	Residual Attention Network (RA-NET)	[53,54]	CNN with Improved Elman Neural Network
[55]	Swin–Transformer and ResNet with Adversarial Domain	[56]	CNN with Improved State Refinement based on GRU
[57]	ResNet34	[58]	CNN with Improved Gated Recurrent Unit (IGRU)
[59]	Few-shot Transfer Learning Siamese ResNet	[60]	Channel Spatial Attention Multi-Scale ResNet (CSA-MResNet)
[61]	Neural Architecture Search Network with Improved Bat Algorithm and Extreme Learning Machine	[62]	CNN and Binarized Neural Networks with Knowledge Distillation
[63–65]	CNN–LSTM	[66]	Convolutional Recurrent Network with Residual Blocks
[67]	RNN with Intra- and Inter-Lead Attention	[68]	Multi-View Multi-Scale Res2Net with Coordinate Attention and Knowledge Distillation
[69]	SE Residual Network (SE-ResNet)	[70]	Attention-based Time-Incremental CNN
[71]	Shortcut Connection CNN–LSTM	[72]	Deep CNN–LSTM with Representation Learning and Sequence Learning
[73]	Custom Faster R-CNN with MobileNetV2	[74]	k-labelsets SE-ResNet-34

[75]	Bi-LSTM with Focal Loss	[76]	Multi-Scale Convolutional Transformer Network
[77]	1D residual U-Net and ResNet34 with Attention	[78]	Multi-Branch CNN–BiLSTM
[79]	AlexNet-1D with Adaptive Threshold Semi-Supervised Learning, Label Generation Process, and a Multi-Branch Network	[80]	CNN–BiGRU with Spatiotemporal Attention
[81]	CNN-based Denoise and Contrast Attention Module	[82]	Masked Time Autoencoder with Transfer Learning
[83]	Hybrid Deep Residual Inception Network and Channel Attention	[84]	CNN–BiGRU with Attention Modules
[85]	Multilevel and Multitask Attention-Based Recurrent Neural Network (MMA-RNN)	[86]	Deep CNN (DCNN)
[87]	CNN-based 1D ShuffleNet	[88]	Semi-Supervised Attention CNN with ECGAugment
[89]	CNN with Modified Elman Neural Network	[90]	CNN–FWS (Feature Weight Screening)

Table S1. End-to-end DL techniques (continued).

Author	Technique	Author	Technique
[91]	CNN and Attention Mechanisms	[92]	CNN-BiLSTM and Attention Mechanism
[93]	CNN with Residual Blocks	[94]	CNN-GRU
[95]	Squeeze-and-Excitation Residual Network with Attention	[96]	CNN and Transformer
[97]	CNN and SSM	[98]	CNN and Attention Mechanism
[99]	CNN-LSTM	[100]	CNN-LSTM
[101]	Temporal and Dilated Convolutional Network	[102]	CNN
[103]	CNN	[104]	CNN, ResNet with a Squeeze-and-Excitation Block, and BiLSTM
[105]	CNN with Mini-InceptionNet	[106]	CNN
[107]	Residual–Temporal Attention and GRU	[108]	CNN and Multi-Head Attention Mechanisms
[109]	Deep CNN	[110]	Deep Multi-Scale Fusion CNN
[111]	CNN-BiLSTM and Attention Mechanisms	[112]	CNN-LSTM
[113]	CNN with Transformer Encoders	[114]	CNN with Attention Mechanism
[115]	CNN-LSTM and Attention Mechanism	[116]	Transformer encoder with Temporal Attention
[117]	CNN, ResNet, and GRU	[118]	CNN-LSTM
[119]	Deep Residual Network and Auto-Encoder	[120]	CNN-based Reinforcement Learning and DNN
[121]	CNN-BILSTM with Contrastive Learning		

Table S2. Abbreviations of cardiac pathologies detected by ECG.

Cardiac pathology	Abbreviation	Cardiac pathology	Abbreviation
2:1 AV Block	2:1_AVB	Complete Right Bundle Branch Block / Right Bundle Branch Block	CRBBB/RBBB
3:1 AV Block	3:1_AVB	Conduction Disturbance	CD
4:1 AV Block	4:1_AVB	Conduction Right	CR
Abnormal QRS Complex	ABQRS	Dilated Cardiomyopathy	DCM
Abnormal Rhythms	AR	Early Repolarization	ER
Abnormalities	ABN	Ectopic Atrial Bradycardia	EAB
Acute Myocardial Infarction	AMI	Ectopic Atrial Rhythm	EAR
Acute Myocardial Infarction/ST Segment Elevation	AMI/STEMI	Ectopic Atrial Tachycardia	EAT
Anterior Injury	AI	Ectopic Right Ventricular	ERV
Anterior Myocardial Infarction	AWMI	First-Degree Atrioventricular Block	IAVB
Anterolateral Infarct	ALMI	Fusion Beat	F
Anterolateral Injury	ALI	Grouped Supraventricular Tachycardia	GSVT
Anteroseptal Infarct	ASMI	Heart Block	HB

Arrhythmias	ARR
Asystole	ASY
Atrial Arrhythmia	AA
Atrial Fibrillation	AF
Atrial Fibrillation or Flutter	AF/AFL
Atrial Flutter	AFL
Atrial Quivering Wave	AQW
Atrial Rhythm Strip	ARS
Atrial Tachycardia	AT
Atrioventricular Node Re-entrant Tachycardia	AVNRT
Atrioventricular Re-entrant Tachycardia	AVRT
AV Block	AVB
Axis Deviation	AD
Beat Of Non-Ventricular Origin	N
Bifascicular Block	BFB
Bigeminal Pattern (Unknown Origin)	BIGU
Bilateral Atrial Hypertrophy	BAH
Bilateral Ventricular Hypertrophy	BVH
Bradycardia	Brady
Bundle Branch Block	BBB
Complete Atrioventricular Block	CAVB
Complete Conduction Right	CCR
Complete Heart Block	CHB
Complete Left Bundle Branch Block	CLBBB
Complete Left Bundle Branch Block/Left Bundle Branch Block	CLBBB/LBBB

Heart Failure	HF
High QRS Voltage	HVOLT
Hypertrophic Cardiomyopathy	HCM
Hypertrophy	HYP
Idioventricular Rhythm	IVR
Incomplete Left Bundle Branch Block	ILBBB
Incomplete Right Bundle Branch Block	IRBBB
Inferior Injury	II
Inferior Myocardial Infarction	IMI
Inferolateral Injury	ILI
Intraventricular Block	IVB
Intrinsic Paroxysmal Atrioventricular Block	IAVB
Ischemic In Anterior Leads	ISCA
Ischemic In Inferior Leads	ISCI
Junctional Arrhythmia	JA
Junctional Bradycardia	JB
Junctional Escape Beat	JEB
Junctional Rhythm	JR
Junctional Tachycardia	JT
Lateral Injury	LI
Lateral Myocardial Infarction	LMI
Left Anterior Fascicular Block	LAFB
Left Anterior/Left Posterior Fascicular Block	LAFB/LPFB
Left Atrial Enlargement	LAE
Left Atrial Hypertrophy	LAH

Table S2. Abbreviations of cardiac pathologies detected by ECG (continued).

**Cardiac pathology**	**Abbreviation**
Left Axis Deviation	LAD
Left Bundle Branch Block	LBBB
Left Fascicular Bundle Branch Block	LFBBB
Left Posterior Fascicular Block	LPFB
Left Ventricular High Voltage	LVHV
Left Ventricular Hypertrophy	LVH
Long PR Interval	LPR
Long QT Interval	LQT
Low QRS Voltage	LQRSV
Low-Voltage QRS In All Leads	LVQRSAL
Low-Amplitude T Waves	LOWT
Multifocal Atrial Tachycardia	MAT
Myocardial Infarction	MI
Paroxysmal Atrial Fibrillation	AFp

**Cardiac pathology**	**Abbreviation**
Sinoatrial Block	SAB
Sinus Arrhythmia	SA
Sinus Atrium To Atrial Wandering Rhythm	SAAWR
Sinus Bradycardia	SB
Sinus Irregularity	SI
Sinus Rhythm/Irregularity	SR/I
Sinus Tachycardia	ST
ST/T Abnormalities	STTA
ST/T Change	STTC
ST-Elevation Myocardial Infarction	STEMI
ST-Segment Depression	STD
ST-Segment Elevation	STE
ST-T Upsloping	STTU
Supraventricular Arrhythmia	SVARR

Paroxysmal Supraventricular Tachycardia	PSVT	Supraventricular Ectopic Beat	SVEB
Pathological Q Waves Present	QWAVE	Supraventricular Tachycardia	SVT
Persistent Atrial Fibrillation	AFf	T Wave Changes	TWC
Poor R Wave Progression	PRWP	T Wave Low and Flat	TWLF
Posterior Myocardial Infarction	PMI	T Wave Overload	TWO
Premature Atrial Contraction	PAC	Third-Degree Atrioventricular Block	3AVB
Premature Atrial Contraction / Supraventricular Premature Beat	PAC/SVPB	Trigeminal Pattern (Unknown Origin)	TRIGU
Premature Complexes	PRC	T Wave Abnormality	TAb
Premature Junctional Complexes	PJC	T Wave Inversion	TInv
Premature Right Intraventricular Excitation	PRIE	Valvular Heart Disease	VHD
Premature Ventricular Contraction	PVC	Variable AV Block	VAVB
Q Wave Abnormal	QAb	Ventricular Arrhythmia	VA
Right Atrial Enlargement	RAE	Ventricular Bigeminy	BIGEMINY
Right Atrial Hypertrophy	RAH	Ventricular Ectopic Beat	VEB
Right Atrial Overload/Enlargement	RAO/RAE	Ventricular Escape Beat	E
Right Axis Deviation	RAD	Ventricular Escape Rhythm	VER
Right Bundle Branch Block	RBBB	Ventricular Fibrillation	VF
Right Ventricular Hypertrophy	RVH	Ventricular Fibrillation Or Flutter	VFF
Right Ventricular Hypertrophy	RHV	Ventricular Flutter Wave	VFW
R-on-T Beat	R	Ventricular Pre-Excitation	VPE
Second-Degree Atrioventricular Block	2AVB	Ventricular Tachycardia	VT
Second-Degree Atrioventricular Block Mobitz Type I	2AVB1	Wandering Atrial Pacemaker	WAP
Second-Degree Atrioventricular Block Mobitz Type II	2AVB2	Wolff–Parkinson–White Syndrome	WPW
Septal Hypertrophy	SEHYP		

Table S3. Cardiac pathology databases used in more than one study.

ID	Database	Considered Pathologies	References
DB01	Chapman–Shaoxing ECG Dataset	AF, AF/AFL, AFL, AMI, AR, AT, AVNRT, AVRT, CAVB, CD, GSVT, SAAWR, SB, SI, SR/I, ST, STTA, SVT	[5,16,22,24,26,34,35,72,76,79,88]
DB02	China Medical University Hospital	2AVB, AF, AFL, AMI/STEMI, BIGEMINY, CHB, EAR, IAVB, PAC, PSVT, PVC, ST	[19,20]
DB03	Chinese Cardiovascular Disease Database (CCDD)	AA, AF, AWMI, BAH, BVH, CRBBB, HB, IAVB, IMI, JA, LAH, LMI, LVH, PAC, PVC, RAH, RVH, SA, SB, ST, TWLF, VA	[18,60]
DB04	China Physiological Signal Challenge 2018 (CPSC-2018)	AF, AF/AFL, CAVB, IPAVB, IAVB, LBBB, PAC, PVC, RBBB, STD, STE, SVT	[4,12–15,19,22,23,28,30–32,35,38,44–47,57,58,68,70,76,78,80,82,82,84]
DB05	CPSC-2021 (V1.0.0)	AF, AFf, AFp	[55,85]
DB06	Creighton University Ventricular Tachyarrhythmia Database (CUDB)	VF, VFW, VT	[1,8]
DB07	European ST-T Database (EDB)	PVC, VF, VT	[8,81]
DB08	Georgia 12-Lead ECG Challenge Database (G12EC)	AF/AFL, AR, CAVB, CD, STTA, SVT	[22,88]

ID	Database	Considered Pathologies	References
DB09	Hefei Hi-tech Cup Dataset (HFHC)	AF, CRBBB, IAVB, LBBB, PAC, PVC, RBBB, SA, SB, ST, STD, STE, TWLF	[60,68]
DB10	St Petersburg INCART 12-Lead Arrhythmia Database (INCART)	F, PVC, SVEB, VEB	[6,81]
DB11	MIT-BIH Arrhythmia Database (MIT-BIH)	AF, AFL, F, LBBB, PAC, PVC, RBBB, SVEB, VEB, VF, VT	[1,2,6–8,10,21,25,40,48,49,51,53,54,58,73,75,81,87]
DB12	MIT-BIH Atrial Fibrillation Database (AFDB)	AF, AFL	[2,25,39,40,47,49,51,53–55,57,59,65,89]
DB13	PhysioNet/CinC Challenge 2017 (CinC2017)	AF	[3,4,9,17,24,28,42,47,52,62,65,66,71,78,83,86]
DB14	PTB Diagnostic ECG Database (PTB)	BBB, DCM, HCM, MI, VHD	[36,50,67]
DB15	PTB-XL	ABQRS, AF, AF/AFL, AFL, AMI, AR, AVB, AWMI, BIGU, CAVB, CD, CLBBB, CRBBB, HVOLT, HYP, ILBBB, IMI, IRBBB, ISCA, ISCI, LAFB/LPFB, LAO/LAE, LBBB, LMI, LOWT, LPR, LQRSV, LQT, LVH, MI, PAC, PMI, PRC, PSVT, PVC, QWAVE, RAO/RAE, RHV, RVH, SA, SB, SEHYP, ST, STTA, STTC, SVARR, SVT, TAb, TInv, TRIGU, WPW	[5,13,22,24,26,30,44,56,61,68,74,76,77,79,82,84,88,90]
DB16	Sejong ECG Dataset	2AVB1, 2AVB2, AF, AF/AFL, CAVB, JR, SVT, VT	[22,24]
DB17	Shaoxing and Ningbo Hospitals (SNH)	2AVB, 2AVB1, 3AVB, AF, AF/AFL, AFL, AQW, AR, ARS, AT, AVB, AVRT, CCR, CD, CR, RVE, IAVB, IVB, JEB, LBBB, LFBBB, LQT, LVH, LVQRSAL, PAC, PRIE, PVC, RAH, RBBB, RVH, SA, SB, SR/I, ST, STD, STE, STTA, STTC, STTU, SVT, TWC, TWO, VEB, VFW, VPE	[14,31,82,88]

Table S4. Cardiac pathology databases used in a single study.

ID	Database	Considered Pathologies	References
DB18	AHA ECG Database (AHA)	E, F, N_(AAMI/AHA), PVC, R, VFF	[87]
DB19	Asan Medical Center Liver Transplant Database	PVC	[81]
DB20	AUMC ICU Biosignal Database	Not specified (unlabeled database used for unsupervised training)	[16]
DB21	Author-collected dataset	AF, CRBBB, ER, IAVB, LAFB, PAC, PVC, TWC	[43]
DB22	Chinese PLA General Hospital	AF	[15]
DB23	CPSC-2018 (public set + CPSC-Extra)	MI	[77]
DB24	CPSC-2020	PVC	[81]
DB25	CPSC-2021 (V1.0.3)	LAD, AF, BBB, AFL, CLBBB, CRBBB, Brady, LAFB, IAVB, IRBBB, SA, Tinv, LQRSV, LBBB, LPR, PAC, PVC, RAD, QAb, RBBB, ST, SB, SVEB, TAb, PRWP, LQT,	[83]
DB26	Custom wearable ECG device recordings	AF	[65]
DB27	Datasets from South Korean University Hospitals	ABN	[33]
DB28	ECG Arrhythmia Classification Dataset	AF, ARR, HF, MI, VER, VF	[63]
DB29	Federal Ministry of Education and Research Dataset	AF	[29]
DB30	First Affiliated Hospital of Nanjing Medical University ECG Database	2AVB, AF, AFL, AMI/STEMI, ASY, IAVB, LBBB, LVH, LVHV, MI, PAC, PVC, RBBB, STTC, TAb, VPE	[11]

ID	Database	Considered Pathologies	References
DB31	First People's Hospital of Guangzhou Database	STEMI	[64]
DB32	Korea University Anam Hospital ECG Dataset	AF, AFL, IAVB, PVC, SB, ST	[69]
DB33	Lobachevsky University Database (LUDB)	MI	[77]
DB34	Long-Term AF Database (LTAFDB)	AF	[59]
DB35	Mayo Clinic ECG Database	2:1_AVB, 2AVB1, 2AVB2, 3:1_AVB, 3AVB, 4:1_AVB, AD, AF, AFL, AI, ALI, ALMI, ASMI, AWMI, BFB, E, EAB, EAR, EAT, IAVB, II, ILI, IMI, IVR, JB, JEB, JR, JT, LAE, LBBB, LI, LMI, LPFB, LVH, MAT, PAC, PJC, PMI, PVC, RAE, RBBB, RVH, SA, SB, ST, SVT, VAVB, VT, WAP	[27]
DB36	MIMIC-III	AF, CRBBB, ER, IAVB, LAFB, PAC, PVC, TWC	[43]
DB37	MIT-BIH Malignant Ventricular Arrhythmia Database (VFDB)	VF, VT	[8]
DB38	MIT-BIH Noise Stress Test Database (NSTDB)	PVC	[81]
DB39	MIT-BIH Supraventricular Arrhythmia Database (SVDB)	PVC	[81]
DB40	Patch Database	AF	[25]
DB41	PhysioNet 2020	AFL, AF, Brady, BBB, PVC, CLBBB/LBBB, QAb, CRBBB/RBBB, IAVB, RAD, Tinv, IRBBB, LAD, LAFB, LPR, LQRSV, PAC/SVPB, SAB, SB, ST, TAb	[37]
DB42	Private 12-lead ECG Dataset	AF	[25]
DB43	QT Database (QTDB)	MI	[77]
DB44	Shandong Provincial Hospital Database (SPHw)[a]	AF	[47]
DB45	Shandong Provincial Hospital Database (SPH)[b]	MI	[77]
DB46	Shandong Provincial Hospital Database (SPHDB)[a]	AF	[39]
DB47	Shanghai Ninth People's Hospital Database (SNPH)	PAC, PVC, SB, ST	[79]
DB48	Shanxi Bethune Hospital Dataset	AF, CRBBB, ER, IAVB, LAFB, PAC, PVC, TWC	[43]
DB49	Telehealth Network Minas Gerais (TNMG)	AF, IAVB, LBBB, RBBB, SB, ST	[14]
DB50	Third Affiliated Hospital of Sun Yat-sen University Database	STEMI	[64]
DB51	Wearable ECG device recordings	AF	[15]
DB52	Wearable long-term ECG device recordings	AF	[55]

[a] Ambulatory ECG, [b] Resting ECG

Table S5. Links to public databases.

ID	Link
DB01	Chapman–Shaoxing ECG Dataset

ID	Link
DB04	CPSC-2018
DB05	CPSC-2021 (V1.0.0)
DB06	CUDB
DB07	EDB
DB08	G12EC
DB10	INCART
DB11	MIT-BIH
DB12	AFDB
DB13	CinC2017
DB14	PTB
DB15	PTB-XL
DB17	SNH
DB18	AHA
DB24	CPSC-2020
DB25	CPSC-2021 (V1.0.3)
DB33	LUDB
DB34	LTAFDB
DB36	MIMIC-III
DB37	VFDB
DB38	NSTDB
DB39	SVDB
DB41	PhysioNet 2020
DB43	QTDB
DB44	SPHw
DB45	SPH
DB46	SPHDB
DB49	TNMG

Table S6. The 14 most studied cardiac pathologies used.

Pathology	References	Count
AF	[1–5,9,11–17,19,20,24,25,27–32,34,35,37–40,42–47,49,51–55,57,59,60,62,63,65,66,68–72,75,76,78–80,82–84,86,89]	63
PVC	[1,4,7,10–12,14,19–21,23,27,28,30–32,35,37,38,43–46,57,58,60,68–70,76,79–84,87]	37
PAC	[1,4,7,10–12,14,19–21,23,27,28,30–32,35,38,43–46,57,60,68,70,76,79,80,82–84]	32
IAVB	[4,11,12,14,19,20,23,27,28,31,32,35,37,38,43–46,57,60,68–70,76,80,82–84]	28
LBBB	[1,4,10–14,23,27,28,30–32,35,38,44–46,57,68,70,76,80,82–84]	26
RBBB	[1,4,10–12,14,23,27,28,30–32,35,38,44–46,57,68,70,76,80,82–84]	25
STD	[4,12,14,23,28,30–32,35,38,44–46,68,70,76,80,82,84]	19
STE	[4,12,14,23,28,30–32,35,38,44–46,68,70,76,80,82,84]	19
AFL	[1,11,16,19,20,27,31,34,37,49,51,53,54,69,72,76,83]	17
MI	[5,11,30,36,44,50,56,61,63,67,68,77,79,82,90]	15
SB	[5,14,16,27,31,34,35,37,60,69,72,76,79,82,83]	15
ST	[14,16,19,20,27,31,34,37,60,69,72,76,79,83]	14
STTC	[5,11,30,31,44,61,68,74,79,82,84,90]	12
SVT	[16,22,27,31,34,35,72,76,79,82]	10

Table S7. Noise and artifact removal—specific techniques.

Techniques and References	Authors' Reported Purpose
[49]: Wavelet	Artifact removal
[5,34]: LOESS	Baseline wander removal
[8]: 0.5-Hz high-pass FIR filter	
[16]: Butterworth filter	
[59]: Sliding window normalization (1-second window size)	

Techniques and References	Authors' Reported Purpose
[77]: Moving average<br>[10]: Wavelet reconstruction	
[15]: 0.5- to 3.5-Hertz elliptical band-pass filter (BPF), order 10<br>[19]: 0.67- to 150-Hertz BPF<br>[26]: Two low-pass filters<br>[44]: 0.5- to 45-Hertz BPF<br>[29]: 5- to 30-Hertz IIR Butterworth BPF<br>[39]: Daubechies wavelet (db6)<br>[43]: Optional BPF<br>[55]: 0.5- to 45-Hertz Butterworth BPF<br>[73]: 0.27- to 45-Hertz Butterworth IIR filter<br>[81]: 0.5- to 40-Hertz Butterworth BPF<br>[89]: 0.5- to 30-Hertz PBF and 50-Hertz Notch filter<br>[72]: 50-Hertz Butterworth low-pass filter, LOESS, and NLM<br>[65]: 0.5- to 45-Hertz Butterworth BPF<br>[88]: 1- to 47-Hertz BPF<br>[90]: BPF	Baseline wander and high-frequency noise removal
[5,77]: NLM<br>[23]: Biorthogonal wavelet (bior2.6)<br>[34]: 50-Hertz Butterworth low-pass filter<br>[40,45,47,48,78,82]: Daubechies wavelet (db6)<br>[42]: None; signals pre-filtered (device-provided)<br>[2,56,64]: Wavelet<br>[21]: Fourier-based smoothing<br>[24,63]: Digital filter<br>[59]: Wiener filter (0.1-second window size)<br>[75]: Savitzky–Golay filter<br>[10]: Wavelet threshold<br>[51]: Hard threshold method<br>[53,54]: Daubechies wavelet (db6)<br>[86]: Daubechies wavelet (dbN)	Noise removal
[34]: NLM	Residual noise removal

Table S8. Preprocessing techniques T02–T05.

Type	Techniques and References
T02	*Z-score:* [1,2,4,6,15,24,29,30,32,39,40,42–45,48,49,51–54,56,59,62,67,68,70,71,76,78,79,81,88,89]<br>*Min–Max*: [3,8,13,17,33,65,69]<br>*Unit variance:* [8]
T03	*Fixed window*: [45]—1.5 s; [2,40,49,51,58,87]—2 s; [75]—3.33 s; [2,54,67,89]—4 s; [48,64]—5 s; [81]—5.12 s; [29]—7 s; [85]—7.5 s; [22]—8 s; [16]—8.2 s; [3,17]—9 s; [14,15,44,55,67,73,79]—10 s; [13]—10.24 s; [39]—15 s; [46]—20 s; [9,12,32,42,59]—30 s<br>*Multiple fixed windows*: [36]—1, 2, and 3 s; [8] —1, 2, 3, and 10 s; [4]—30 and 60 s<br>*Overlapping sliding windows*: [23,66,78]
T04	*Downsampling*<br>[8,28,37,45,68,76]: 100 Hz;<br>[78]: 120 Hz;<br>[67]: 125 Hz;<br>[29,59]: 128 Hz;<br>[39]: 200 Hz;<br>[79]: 204.8 Hz;<br>[2,4,16,40,49,51,54,56,58,67,81,82,84]: 250 Hz;<br>[80]: 256 Hz;<br>[13,14,57]: 400 Hz.<br>*Upsampling via linear interpolation:*<br>[25]—256 Hz; [33]—200 Hz

Table S8. Preprocessing techniques T02–T05 (cont.).

Type	Techniques and References
T05	[13]: Trimmed or zero-padded to 4,096 samples<br>[30]: Zero-padding to 30 s and cropping to 30 s<br>[32]: Zero-padding to 30 s<br>[42]: Expansion to 30 s by replication and 30 s random cropping<br>[46]: Records that are too short are replicated to the specified length, while those that are too long are cropped<br>[4]: Zero-padding to 30 s and 60 s depending on the database<br>[12]: Zero-padding and trimming to 30 s<br>[9]: If a record has more than 9,000 samples, it is divided into 9,000-sample segments with 50% overlap; if it has fewer, it is concatenated with itself until reaching 9,000 samples<br>[14]: Zero-padding to 10 s<br>[31]: Cropping or padding to 10 s<br>[35]: Length adjustment to 10 s, trimming longer signals and zero-padding shorter ones<br>[37]: Cropping records to 2.5 s<br>[43]: Cropping to 8,192 points and zero-padding if short<br>[52]: Segmentation and merging, copying, and extracting to 4,500 samples<br>[63]: Resizing and filling<br>[71]: Longer data cropping and shorter data deleting into 5-, 10-, and 20-second segments<br>[83]: Cropping to 2 minutes<br>[60]: Middle 10-second segment selected; shorter data discarded<br>[62]: Bucket padding<br>[68]: Cropping and oversampling to 10 s<br>[70]: Zero-padding to 60 s<br>[76]: Padding and upsampling<br>[80]: Cropping and zero-padding to 60 s<br>[82]: Resampling to 10 s using FFT and overlapping sliding windows<br>[84]: Cropping or zero-padding to 60 s<br>[88]: Zero-padding to 12.288 s

Table S9. Preprocessing techniques T06–T12.

Type	Techniques and References
T06	[3]: ADYSAN, Borderline-SMOTE, SMOTE<br>[17]: Segmentation into 9-second windows with overlap<br>[34]: Class balancing: SMOTE–Tomek algorithm<br>[8]: Downsampling of majority classes<br>[6]: Downsampling of majority class and upsampling of minority classes<br>[12]: Class replication<br>[48]: Overlapping segmentation<br>[50]: GAN<br>[71]: Discarding noisy data and rhythms not used in the study, and adding replicas of the target category<br>[73]: SMOTE<br>[75]: Oversampling and focal loss<br>[87]: Stepped window sampling<br>[11]: Data amplification
T07	[3]: Clipping<br>[5]: Removal of missing samples<br>[17]: Removal of noisy beginnings<br>[20]: Removal of duplicates<br>[23]: Replacement of anomalous values based on a threshold<br>[32]: Removal of NaN data<br>[34]: Removal of zero and missing data<br>[22]: Removing 1-second segments from the beginning and end of each 10-second recording due to artifacts; in external databases, extracting an 8-second middle segment<br>[24]: Trimming 1 s from the ends of each 10-second ECG record<br>[59]: Removal of noisy segments<br>[73]: Removal of the initial 10 s of each record<br>[60]: Discarding the initial 1.25 s
T08	[28]: Random cropping with Daubechies-4 and Daubechies-6 wavelets<br>[32]: Scaling, jittering, time warping, magnitude warping, random sampling, and permutation

Type	Techniques and References
	[35]: DropLead augmentation technique, applied during training, randomly omits one of three input signals<br>[37]: Semantic split-join and weighted peak noise smoothing were applied as semantic transformations<br>[41]: Heart Disease Prediction GAN (model proposed by the author)<br>[45]: Slicing variable-length windows in signals >30 s and extracting random segments of 6 to 15 s<br>[59]: 1st- or 2nd-order high-pass Butterworth filter tuned to 1-Hertz corner frequency<br>[63]: Data augmentation to prevent overfitting<br>[67]: Insertion of random periodic spikes, additive white Gaussian noise, and random baseline flips<br>[66]: Neural transfer network<br>[70]: Temporal scaling and 1.5-second zero masking
T09	[47]
T10	[60,68]
T11	[17,57]
T12	[65]: FFT and 1- to 45-Hertz Hanning window-based filter

Table S10. PRISMA 2020 for Abstract Checklist.

Topic	No.	Item	Reported?
TITLE			
**Title**	1	Identify the report as a systematic review.	Yes
BACKGROUND			
**Objectives**	2	Provide an explicit statement of the main objective(s) or question(s) the review addresses.	Yes
METHODS			
**Eligibility criteria**	3	Specify the inclusion and exclusion criteria for the review.	Yes
**Information sources**	4	Specify the information sources (e.g. databases, registers) used to identify studies and the date when each was last searched.	Yes
**Risk of bias**	5	Specify the methods used to assess risk of bias in the included studies.	Yes
**Synthesis of results**	6	Specify the methods used to present and synthesize results.	Yes
RESULTS			
**Included studies**	7	Give the total number of included studies and participants and summarise relevant characteristics of studies.	Yes
**Synthesis of results**	8	Present results for main outcomes, preferably indicating the number of included studies and participants for each. If meta-analysis was done, report the summary estimate and confidence/credible interval. If comparing groups, indicate the direction of the effect (i.e. which group is favoured).	Yes
DISCUSSION			
**Limitations of evidence**	9	Provide a brief summary of the limitations of the evidence included in the review (e.g. study risk of bias, inconsistency and imprecision).	Yes
**Interpretation**	10	Provide a general interpretation of the results and important implications.	Yes
OTHER			
**Funding**	11	Specify the primary source of funding for the review.	Yes
**Registration**	12	Provide the register name and registration number.	Yes

Table S11. PRISMA 2020 Checklist.

Section and Topic	Item #	Checklist item	Location where item is reported
**TITLE**			
Title	1	Identify the report as a systematic review.	Pg 1
**ABSTRACT**			
Abstract	2	See the PRISMA 2020 for Abstracts checklist.	
**INTRODUCTION**			
Rationale	3	Describe the rationale for the review in the context of existing knowledge.	Pg 1-3
Objectives	4	Provide an explicit statement of the objective(s) or question(s) the review addresses.	Pg 3, 10
**METHODS**			
Eligibility criteria	5	Specify the inclusion and exclusion criteria for the review and how studies were grouped for the syntheses.	Pg 7-8
Information sources	6	Specify all databases, registers, websites, organisations, reference lists and other sources searched or consulted to identify studies. Specify the date when each source was last searched or consulted.	Pg 7-9
Search strategy	7	Present the full search strategies for all databases, registers and websites, including any filters and limits used.	Pg 7
Selection process	8	Specify the methods used to decide whether a study met the inclusion criteria of the review, including how many reviewers screened each record and each report retrieved, whether they worked independently, and if applicable, details of automation tools used in the process.	Pg 8
Data collection process	9	Specify the methods used to collect data from reports, including how many reviewers collected data from each report, whether they worked independently, any processes for obtaining or confirming data from study investigators, and if applicable, details of automation tools used in the process.	Pg 8
Data items	10a	List and define all outcomes for which data were sought. Specify whether all results that were compatible with each outcome domain in each study were sought (e.g. for all measures, time points, analyses), and if not, the methods used to decide which results to collect.	Pg 8
	10b	List and define all other variables for which data were sought (e.g. participant and intervention characteristics, funding sources). Describe any assumptions made about any missing or unclear information.	Pg 7-8

Section and Topic	Item #	Checklist item	Location where item is reported
Study risk of bias assessment	11	Specify the methods used to assess risk of bias in the included studies, including details of the tool(s) used, how many reviewers assessed each study and whether they worked independently, and if applicable, details of automation tools used in the process.	Pg 7
Effect measures	12	Specify for each outcome the effect measure(s) (e.g. risk ratio, mean difference) used in the synthesis or presentation of results.	Not applicable
Synthesis methods	13a	Describe the processes used to decide which studies were eligible for each synthesis (e.g. tabulating the study intervention characteristics and comparing against the planned groups for each synthesis (item #5)).	Pg 7-8
	13b	Describe any methods required to prepare the data for presentation or synthesis, such as handling of missing summary statistics, or data conversions.	Pg 7-8
	13c	Describe any methods used to tabulate or visually display results of individual studies and syntheses.	Pg 7-8
	13d	Describe any methods used to synthesize results and provide a rationale for the choice(s). If meta-analysis was performed, describe the model(s), method(s) to identify the presence and extent of statistical heterogeneity, and software package(s) used.	Pg 7-8
	13e	Describe any methods used to explore possible causes of heterogeneity among study results (e.g. subgroup analysis, meta-regression).	Not applicable
	13f	Describe any sensitivity analyses conducted to assess robustness of the synthesized results.	Not applicable
Reporting bias assessment	14	Describe any methods used to assess risk of bias due to missing results in a synthesis (arising from reporting biases).	Pg 7-8
Certainty assessment	15	Describe any methods used to assess certainty (or confidence) in the body of evidence for an outcome.	Not applicable
**RESULTS**			
Study selection	16a	Describe the results of the search and selection process, from the number of records identified in the search to the number of studies included in the review, ideally using a flow diagram.	Pg 9, Figure 7
	16b	Cite studies that might appear to meet the inclusion criteria, but which were excluded, and explain why they were excluded.	Pg 9, Figure 7
Study characteristics	17	Cite each included study and present its characteristics.	Tables 5-14

Section and Topic	Item #	Checklist item	Location where item is reported
Risk of bias in studies	18	Present assessments of risk of bias for each included study.	Not applicable
Results of individual studies	19	For all outcomes, present, for each study: (a) summary statistics for each group (where appropriate) and (b) an effect estimate and its precision (e.g. confidence/credible interval), ideally using structured tables or plots.	Pg 25-31
Results of syntheses	20a	For each synthesis, briefly summarise the characteristics and risk of bias among contributing studies.	Pg 25-31
	20b	Present results of all statistical syntheses conducted. If meta-analysis was done, present for each the summary estimate and its precision (e.g. confidence/credible interval) and measures of statistical heterogeneity. If comparing groups, describe the direction of the effect.	Pg 25-31
	20c	Present results of all investigations of possible causes of heterogeneity among study results.	Not applicable
	20d	Present results of all sensitivity analyses conducted to assess the robustness of the synthesized results.	Not applicable
Reporting biases	21	Present assessments of risk of bias due to missing results (arising from reporting biases) for each synthesis assessed.	Not applicable
Certainty of evidence	22	Present assessments of certainty (or confidence) in the body of evidence for each outcome assessed.	Not applicable
**DISCUSSION**			
Discussion	23a	Provide a general interpretation of the results in the context of other evidence.	Pg 31-36
	23b	Discuss any limitations of the evidence included in the review.	Pg 37
	23c	Discuss any limitations of the review processes used.	Pg 37
	23d	Discuss implications of the results for practice, policy, and future research.	Pg 31-36
**OTHER INFORMATION**			
Registration and protocol	24a	Provide registration information for the review, including register name and registration number, or state that the review was not registered.	Not registered
	24b	Indicate where the review protocol can be accessed, or state that a protocol was not prepared.	Pg 7
	24c	Describe and explain any amendments to information provided at registration or in the protocol.	Not applicable

Section and Topic	Item #	Checklist item	Location where item is reported
Support	25	Describe sources of financial or non-financial support for the review, and the role of the funders or sponsors in the review.	Not applicable
Competing interests	26	Declare any competing interests of review authors.	Pg 36
Availability of data, code and other materials	27	Report which of the following are publicly available and where they can be found: template data collection forms; data extracted from included studies; data used for all analyses; analytic code; any other materials used in the review.	Not applicable
